# Cortical activity for conversational responses in young neurotypical individuals, older neurotypical individuals, and individuals with aphasia: A functional near-infrared spectroscopy study

**DOI:** 10.1162/IMAG.a.1125

**Published:** 2026-02-10

**Authors:** Emily J. Braun, Erin A. Carpenter, Yuanyuan Gao, Meryem A. Yücel, David A. Boas, Swathi Kiran

**Affiliations:** Boston University Center for Brain Recovery, Boston, MA, United States; Boston University Neurophotonics Center, Boston, MA, United States

**Keywords:** functional near-infrared spectroscopy, fNIRS, language, aphasia, neuroimaging

## Abstract

Functional near-infrared spectroscopy (fNIRS) has the potential for evaluating language and conversational interaction in an ecologically valid way given that participants can be seated and interacting with a conversational partner. Aphasia, an acquired language disorder, impacts language abilities, including conversational interaction. Understanding of cortical activity in people with aphasia for conversational tasks could provide an ecologically valid index of brain function and recovery patterns in this population. This study provides pilot data in the use of fNIRS to evaluate language production for conversational responses in young (adult) neurotypical individuals, adults with post-stroke aphasia, and age-matched (i.e., older) neurotypical adults. Experiment 1 evaluated cortical activity for conversational responses versus sentence repetition in a computer-based conversational question task. Experiment 1 results showed differentiation between experimental conditions (with greater fronto-temporoparietal cortical activity for question answering vs. repetition) in young healthy individuals that was not seen in the people with aphasia and age-matched individuals. Experiment 2 evaluated cortical activity for conversational responses in a structured conversational question task with a live interlocutor versus sentence repetition. Experiment 2 results showed lower cortical activity across regions of interest in the age-matched group and greater cortical activity for question answering versus repetition in bilateral temporal and left parietal regions of interest in the people with aphasia. This study provides preliminary evidence for the potential of fNIRS to characterize cortical activity after stroke for functional language tasks.

## 1 Introduction

More than 9.4 million adults in the United States have experienced a stroke (Martin et al., 2024), with approximately 30% of ischemic or ischemic–hemorrhagic strokes resulting in aphasia (Flowers et al., 2016). Aphasia, an acquired language disorder after brain injury, has long-term negative consequences on social relationships, quality of life, and return to previous home and community responsibilities.

### 1.1 Use of functional near-infrared spectroscopy (fNIRS) to evaluate cortical activity for language and communication

In recent decades, there has been significant interest in understanding the neural mechanisms supporting language and communication performance and recovery in individuals with post-stroke aphasia, with the goal of better characterizing the nature of post-stroke aphasia and developing more robust prognosis and individualized, effective treatment approaches. Much of the existing neuroimaging research for aphasia has focused on single word production (e.g., naming pictures), most often completed in a scanner with functional magnetic resonance imaging (fMRI) (LaCroix et al., 2021; Wilson & Schneck, 2021). However, in the real world, communication involves connected speech in a range of contexts and tasks which have yet to be fully investigated at the neural level, in part because they are difficult to examine in an MRI scanner given the sensitivity to motion artifacts, supine position of participants, and high acoustic noise.

FNIRS has the potential to address questions in cognitive neuroscience for clinical populations that cannot be fully investigated with fMRI. Compared with fMRI, fNIRS is portable, less susceptible to motion artifacts, more comfortable (i.e., can be done sitting up comfortably rather than in an enclosed scanner), and lacks the acoustic noise of fMRI (Quaresima & Ferrari, 2019). All of these benefits give fNIRS the potential for ecologically valid data collection for language and communication in a range of contexts, including conversational interaction (Suda et al., 2010). FNIRS has been successfully used to investigate speech and language in neurotypical individuals (Dieler et al., 2012) and clinical populations (Butler et al., 2020).

### 1.2 Use of fNIRS to evaluate language processing in neurotypical individuals

In neurotypical adults, cortical activation for discourse (i.e., “a unit of language longer than a single sentence”; Dipper et al., 2021) has been evaluated via fNIRS and generally has shown greater frontotemporal activity for discourse production when compared with reading, listening, or repetition (Cannizzaro et al., 2016; Suda et al., 2010). To evaluate discourse production in dialogue, Suda and colleagues developed a paradigm evaluating production in conversation in 30 neurotypical speakers of Japanese. The goal of this work was to develop a paradigm for evaluating social interaction and conversation in an ecologically valid manner that could be used in neurotypical and patient populations. In this paradigm, participants were seated across the table from a conversational partner and were asked to have a conversation about food in 15-second increments (Suda et al., 2010). In the control condition, participants repeated syllables in 15-second increments. In this example, while discourse production and repetition both involve linguistic and motor output demands, discourse production has spontaneous formulation demands not needed for repetition. The control condition accounts speech production demands and allows for better isolation of spontaneous language formulation demands. This paradigm was novel in that it evaluated interactions between two live interlocutors. In these neurotypical individuals, the authors found greater activation in frontotemporal channels for conversation versus syllable repetition. They have since applied this paradigm to multiple clinical populations (Suda et al., 2011; Takei et al., 2013, 2014). This paradigm provides a methodological starting point for other studies evaluating cortical activity during conversation involving spontaneous responses via fNIRS, including the current investigation.

### 1.3 Use of fNIRS to evaluate language processing in individuals with aphasia

There has been increased use of fNIRS for investigation of post-stroke brain function. However, these studies have largely focused on motor recovery (Yang et al., 2019). A limited number of studies have used fNIRS to evaluate task-based cortical activity for language and other cognitive tasks in individuals with aphasia (Chang et al., 2022; Gilmore et al., 2021; Hara et al., 2017; H. Li et al., 2023; Sakatani et al., 1998). In this small set of studies, the most common experimental task was confrontation naming of pictured nouns with a baseline or rest phase as a control condition. For example, Sakatani and colleagues used fNIRS to investigate cortical activation for speech and language tasks in 10 individuals with nonfluent aphasia (including a mix of acute, sub-acute, and chronic aphasia), 13 age-matched controls, and 6 individuals who were post-stroke without aphasia (Sakatani et al., 1998). Participants completed 3 tasks: confrontation naming, counting from 1 to 50, and talking about what happened yesterday. The authors reported individual-level results showing a variety of different response patterns, with the most common pattern in the individuals with aphasia being an increase in oxygenated (HbO) and deoxygenated (HbR) hemoglobin in the left prefrontal cortex during confrontation naming and narrative discourse as compared with baseline.

Gilmore and colleagues examined cortical activation for picture naming via fNIRS in 24 young healthy individuals, 17 older healthy individuals, and 6 individuals with chronic post-stroke aphasia (Gilmore et al., 2021). Participants named pictured nouns (experimental condition) or said “skip” when a scrambled picture appeared (control condition). While results were variable, overall individuals with aphasia showed activation differences between experimental and control conditions in fewer regions of interest (ROIs) than the healthy individuals. In contrast, Li and colleagues found greater HbO in individuals with chronic post-stroke global aphasia versus controls in left-hemisphere language regions (left inferior frontal gyrus (IFG), middle temporal gyrus (MTG), superior temporal gyrus (STG), premotor cortex, and supplementary motor area) for picture naming. The authors attribute this to “overcompensation.” These contrasting results highlight two potential hypotheses relating to cortical activity for language in individuals with post-stroke aphasia: (1) decreased cortical activity due to the stroke lesions versus (2) increased cortical activity due to compensation and/or cognitive effort required for task completion.

The current study aimed to evaluate cortical activity during conversational responses requiring spontaneous formulation via fNIRS for young neurotypical individuals, individuals with post-stroke aphasia, and age-matched control participants. To our knowledge, no previous studies have evaluated cortical activity via fNIRS during functional conversational tasks in people with aphasia.

### 1.4 Research questions

1. In three groups (young neurotypical individuals, individuals with post-stroke aphasia, and age-matched neurotypical individuals), can fNIRS index cortical activity differences between language formulation (i.e., conversational responses) and sentence repetition?
  Hypothesis: All groups (young neurotypical, individuals with post-stroke aphasia, and age-matched neurotypical individuals) will show greater cortical activity (via greater HbO) during discourse production as compared with sentence repetition in bilateral frontotemporal regions but not in control regions, bilateral precentral gyri (PCG). Bilateral parietal ROIs will be treated as additional ROIs given their known involvement in language processing.
2. Are there differences in cortical activity for conversational responses and sentence repetition among young neurotypical individuals, individuals with post-stroke aphasia, and age-matched neurotypical individuals?
  Hypothesis: Individuals with post-stroke aphasia will show less cortical activity (via lower HbO) during discourse production in bilateral frontotemporal regions when compared with age-matched neurotypical participants based upon fMRI evidence of lower cortical activity for language processing, particularly in the left hemisphere, for individuals with aphasia compared with neurotypical controls (Wilson & Schneck, 2021). Bilateral parietal ROIs will be treated as additional ROIs given their known involvement in language processing.

## 2 Ethics

This study was approved by the institutional review board at Boston University (IRB Protocol Numbers 3309E and 4966E) and written informed consent was obtained from all participants for being included in the study.

## 3 Experiment 1 Methods

### 3.1 Participants

Young neurotypical individuals were recruited from a university setting. Age-matched neurotypical participants were recruited by contacting individuals from an existing database in the Boston University Center for Brain Recovery and through self-referral to the Center for Brain Recovery. Individuals with aphasia were recruited from an existing database in the Center for Brain Recovery as well as through contacting local clinicians, aphasia groups, and community groups. All individuals with aphasia met the following inclusion criteria: (1) chronic (i.e., greater than 6 months) post-stroke aphasia due to a left-hemisphere stroke with presence of aphasia determined by clinical diagnosis in the medical record, (2) aphasia severity moderate or better (i.e., Western Aphasia Battery – Revised Aphasia Quotient ≥ 50; Kertesz, 2007) given the task demand, and (3) no significant neurological or psychiatric history prior to stroke besides anxiety and/or depression. Given the prevalence of anxiety and depression in the general population and depression in the post-stroke population specifically (J.-M. Li et al., 2025), participants with anxiety and/or depression were not excluded from the study across groups. Eligibility was determined by participant history questionnaires (all participants), review of medical records requested from participants (individuals with aphasia), and behavioral assessments (individuals with aphasia and age-matched neurotypical adults). A pure tone audiometric screening was performed at 30 dB HL at 500, 1000, 2000, and 4000 Hz for the individuals with aphasia and the age-matched neurotypical individuals given the possibility of age-related hearing loss. For individuals who failed the hearing screening, increased volume during all study tasks was offered and the experimenter verified with the participants whether they perceived the volume was adequate.

In Experiment 1, participants were 15 young neurotypical adults (mean age = 23.5 years, SD = 4.1; 7 female, 8 male), 16 individuals with chronic post-stroke aphasia (mean age = 61.3 years, SD = 9.3; 4 female, 12 male), and 15 age-matched neurotypical adults (mean age = 59.5 years, SD = 9.6; 9 female, 6 male; see Table 1 for young and age-matched demographics and Table 2 for individuals with aphasia demographics). Three young participants reported a history of depression. One age-matched participant reported a history of dyscalculia as a child. For the hearing screen, 13 of 15 age-matched participants passed at all frequencies in at least one ear and 11 of 16 people with aphasia passed the hearing screening at all frequencies in at least one ear. All age-matched neurotypical participants scored within normal limits on the Mini Mental State Examination, 2nd Edition Expanded Version (Folstein & Folstein, 2010) as a cognitive screening (average t-score = 56, standard deviation = 6, range = [46, 63]).^1^

**Table 1.** Experiment 1 young and age-matched neurotypical demographics.

ID	Age	Sex	Handedness	Race	Ethnicity	Native language	Other languages	Years educ.
Y1	20	M	R	White	NH	English	None	14
Y2	20	M	R	White	H	English	Spanish	14
Y3	35	F	R	White	NH	English	Russian	20
Y4	25	M	R	White	NH	English	French*	19
Y5	19	F	R	Asian	NH	English	Korean	13
Y6	28	F	R	White	NH	English	Italian*	19
Y7	21	F	R	Asian	NH	English	Korean	15
Y8	24	F	R	White	NH	English	-	18
Y9	21	M	R	Asian	H	English	Spanish	13
Y10	26	F	R	Black	NH	Igbo	English	20
Y11	23	F	R	Black	NH	English, Igbo	-	17
Y12	23	M	R	White	H	English	Spanish	16
Y13	24	M	Ambidextrous	Black	NH	English	French, Patois	15
Y14	23	M	R	Asian	NH	English	Urdu	18
Y15	20	M	R	White	H	English	Spanish, Danish, Japanese	15
AM1	59	M	R	White	NH	English	-	16
AM2	59	M	R	White	NH	English	-	16
AM3	65	F	R	White	NH	English	-	17
AM4	63	F	R	White	NH	English	French*	16
AM5	63	F	R	White	NH	English	Spanish, French	17
AM6	72	F	R	White	NH	English	-	16
AM7	51	M	R	White	NH	English	-	16
AM8	52	M	R	White	NH	Portuguese	English, Spanish	12
AM9	55	M	R	Black	NH	English	-	16
AM10	46	M	R	White	NH	English	Spanish, French, Portuguese, Arabic	16
AM11	58	F	R	Black	NH	English	-	12
AM12	69	F	R	White	NH	English	-	18
AM13	45	F	R	White	NH	English	-	14
AM14	80	F	R	White	NH	English	-	19
AM15	56	F	R	White	NH	English	-	17

* Basic or intermediate.

Y: young; AM: age-matched; NH: non-Hispanic; H: Hispanic; Educ: education; M: male; F: female.

**Table 2.** Experiment 1 demographics for people with aphasia.

ID	Age	Sex	Hand	Race	Ethnicity	Native language	Other languages	Years educ.	MPO	Stroke location (per medical record)
A1	69	M	L	White	NH	English	-	17	70	Left MCA (fronto-temporoparietal)
A2	62	M	R	White	NH	English	Spanish	19	21	Left MCA (parietal)
A3	57	M	R	Black	NH	English	-	12	152	Left MCA (frontotemporal, subcortical)
A4	57	M	R	White	NH	English	-	12	162	Left MCA
A5	74	F	R	White	NH	English	-	16	26	Left hemisphere (temporal, subcortical)
A6	75	M	R	White	NH	English	-	20	84	Left MCA
A7	66	M	R	Black	NH	English	-	12	44	Left MCA (temporal)
A8	69	M	R	White	NH	English	-	16	57	Left MCA
A9	60	F	R	White	NH	English	Spanish	17	154	Left hemisphere (frontoparietal)
A10	60	M	L	White	NH	English	-	12	299	Left hemisphere
A11	58	F	R	Black	NH	English	-	16	73	Left hemisphere*
A12	57	M	R	White	NH	English	-	16	16	Left hemisphere (thalamus, basal ganglia)
A13	68	M	R	White	NH	English	-	16	244	Left hemisphere (temporoparietal)
A14	36	F	R	White	NH	English	Spanish	18	8	Left hemisphere
A15	54	M	R	White	NH	English	-	18	127	Left MCA
A17	58	M	R	White	NH	English	-	16	53	Left MCA
Mean	61.3							15.8	99.4	
SD	9.3							2.6	84.4	

* History of multiple strokes.

M: male; F: female; Hand: handedness; R: right; L: left; H: Hispanic; NH: non-Hispanic; MPO: months postonset.

### 3.2 Behavioral assessment

Standardized behavioral assessment of individuals with aphasia was completed to characterize participants’ impairment. To assess cognitive-linguistic skills, individuals with aphasia completed the Western Aphasia Battery – Revised Part 1 (Kertesz, 2007), a measure of aphasia severity assessing language skills and the Cognitive Linguistic Quick Test – Plus, a measure of linguistic and nonlinguistic cognitive skills (Helm-Estabrooks, 2017). Performance on the picture description subtest of the WAB-R AQ was characterized via global coherence, a measure of overall adherence to the topic (Harris Wright & Capilouto, 2012), and type-token ratio, a measure of lexical diversity (Fromm et al., 2017). Motor speech was characterized by the Apraxia of Speech Rating Scale 3.5 (Duffy et al., 2023).

### 3.3 Experimental task development

Open-ended questions for the task were generated by a group of five fluent English-speaking research assistants from the Center for Brain Recovery. These questions were then curated by the first author and modified for style and length with consultation from the research team. Questions were narrowed down to 18 *Personal* and 18 *General* with an attempt to balance question topic and length between runs. These questions and the control sentence were then video- and audio-recorded by a young adult female speaker of standard American English in a quiet room using a Logitech webcam and the freely available Open Broadcaster Software on a Windows desktop computer. The speaker used a conversational tone and neutral affect. The speaker was visible from the shoulders up with no hand gestures visible. Each stimulus item was recorded as a separate mp4 file. Video editing was done using Apple iMovie software. Recorded stimuli were edited to be 5.5 seconds in length each with speech beginning 0.1 seconds after the video onset. The behavioral task was programmed in PsychoPy3 2021.1.2, which allows for time locking of experimental events to fNIRS data via LabStreamingLayer triggers (Peirce et al., 2019). The behavioral task was then piloted with three fluent English speakers. Slight adjustments to timing and computer screen lighting were made based on pilot participant feedback.

### 3.4 Experimental task design

**Fig. 1.**
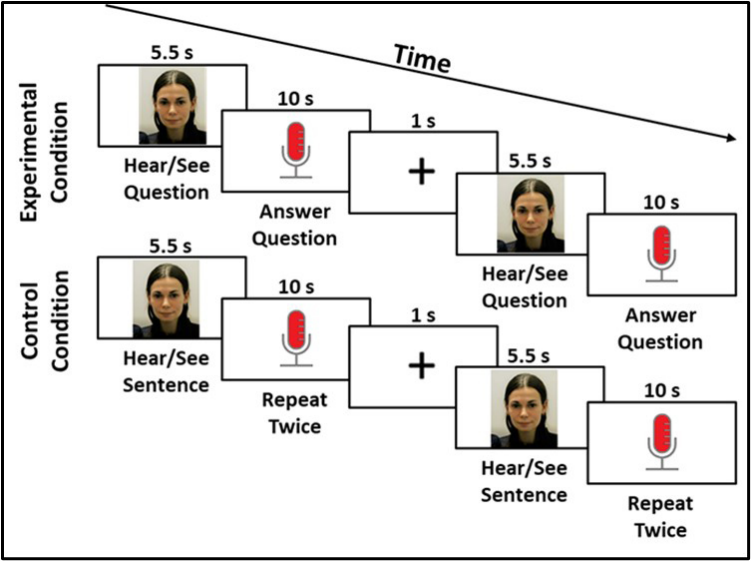
Experiment 1 task design.

**Appendix Fig. A1.**
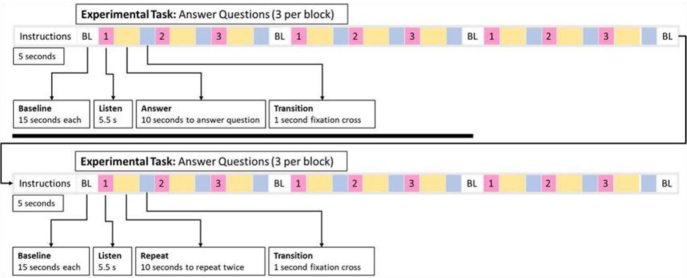
Experiment 1 task timing.

Auditory information was presented using a Jabra speaker and verbal responses were recorded using a Jabra microphone. The Experiment 1 task consisted of four runs of 7 minutes and 7 seconds each (see Fig. 1 for Experiment 1 task design and Appendix Fig. A1 for Experiment 1 task timing). During the task, participants answered conversational questions (experimental condition, e.g., *What do you like to do in the summertime?*) or repeated a sentence twice (control condition; *The sun is shining and the birds are out*). Each stimulus item was presented in an audio–visual clip (i.e., video recording of a woman asking an experimental question or saying the control sentence); each response period was 10 seconds long; and there was a 1-second fixation cross presented after each response period in a block. During the response period, participants saw a progress bar on the screen to allow them to gauge the remaining time to answer.

Stimulus items were presented in three-question (experimental condition) or three-sentence (control condition) blocks of 48.5 seconds with a 15-second baseline (with fixation cross presentation) between blocks and at the beginning and end of each run. Experimental blocks were either *General* questions related to social, cultural, and world knowledge (e.g., *What do plants need to survive?*) or *Personal* questions related to the individual’s life (e.g., *What do you like to do to relax?*; see Supplementary Material for list of questions). Each of the 4 7-minute runs contained 3 experimental blocks and 3 control blocks (18 general questions, 18 personal questions, 36 experimental questions total). Two of the four runs contained experimental blocks with *Personal* questions and two of the four runs contained experimental blocks with *General* questions. To mitigate order effects, question order was randomized for every question block and condition and run order were counterbalanced across participants. Participants completed a practice task after receiving verbal instructions about the task. The practice task included one block of General questions (three questions in the block), one block of Personal questions (three questions in the block), and one block of control sentence repetition (three stimulus items in the block).

Experiment 1 was first completed with the young neurotypical group and then modified to accommodate individuals with aphasia, which included (1) additional visual representation of the experimental conditions with the task instructions; (2) increased pause time (controlled by the experimenter); (3) the experimenter reading aloud the questions for individuals with more severe aphasia prior to each run to reduce formulation demands (N = 3); and (4) providing participants with more severe production impairment with choral cues (i.e., speaking aloud with the clinician) during the repeat condition as needed to promote greater accuracy (N = 6).

### 3.5 Evaluation of task performance

The PsychoPy experiment was programmed to automatically record and save responses in each period separately for later review. Audio recordings were later broadly transcribed by the first author and seven trained research assistants. During transcription, the individual transcribing made a manual marking rounded to the nearest second at the end of the participant response in each block. The talk time annotations at the end of talking in each block were then averaged within each participant to create two values per participant for this experiment (one value for each condition). These values were then used as regressors in statistical models to account for varying talk times across conditions and participants (see Appendix A2 for sample response and Table 3 for talk time average values across groups and conditions for Experiment 1).

**Table 3.** Average end of talking time per trial Experiment 1.

Group	Questions	Repeat
Young	7.5 (1.29)	6.2 (.94)
Age-matched	8.2 (1.22)	7.3 (.71)
People with Aphasia	7.5 (1.1)	6.5 (1.5)
Overall	7.7 (1.2)	6.7 (1.2)

Mean (standard deviation) in seconds (each trial is 10 seconds in length).

### 3.6 fNIRS probe design

**Fig. 2.**
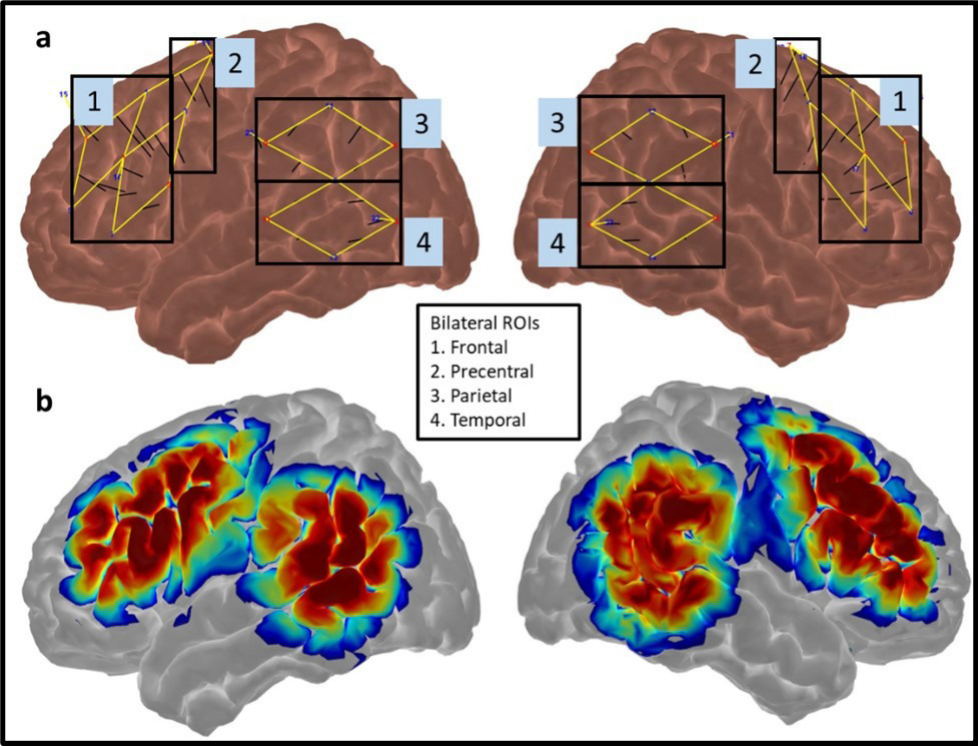
fNIRS probe design. (a) fNIRS probe design in AtlasViewer overlayed on template cortex with markers indicating channel data averaged to create an ROI and (b) fNIRS probe sensitivity profile in AtlasViewer, with warmer colors indicating higher sensitivity to cortex.

The experiment used an NIRx NIRSport2 (NIRx Medical Technologies, Germany) with 16 sources and 16 detectors with 1 detector occupied by 8 short-distance detectors. The fNIRS probe was designed in AtlasViewer (Aasted et al., 2015). The fNIRS probe consisted of 37 long separation measurement channels as well as 8 short separation channels used to regress out signal from the scalp unrelated to the neural signal (Yücel et al., 2015). The fNIRS probe was designed to gather data from ROIs active during language processing in neurotypical individuals and individuals with aphasia based on fMIRI (Friederici, 2011; Price, 2012; Wilson & Schneck, 2021) and fNIRS studies (Gilmore et al., 2021). Primary ROIs were bilateral frontal (middle frontal gyrus (MFG) and inferior frontal gyrus (IFG)) and bilateral temporal (MTG and STG). Bilateral PCG were considered control ROIs, with similar activity expected for both conditions given similar speech production demands. Bilateral parietal ROIs (angular gyrus (AG) and supramarginal gyrus (SMG)) were considered exploratory ROIs. One asymmetric channel in the left MFG was not analyzed given that there was no corresponding right-hemisphere channel (see Fig. 2 for fNIRS probe design and Supplementary Material for channel MNI coordinates).

### 3.7 fNIRS data collection

Data collection took place in a relatively quiet, well-lit room with participants seated at a computer screen. Set-up included (1) prior to cap placement, head measurements were taken to ensure proper cap size selection and for use in later analyses. Head measurements included head circumference, distance from nasion to inion, and distance from left preauricular to right preauricular points; (2) following this, cap placement and adjustment was made to promote the best possible signal quality with adequate scalp to optode coupling; (3) after initial calibration, a shower cap was placed on the participant’s head to reduce effects of ambient light on the detectors; and (4) an additional check for signal quality was completed with adjustments as needed prior to beginning the tasks.

In-home assessment was offered to individuals with aphasia if they reported difficulty traveling to the laboratory, typically due to physical disability, distance from the laboratory, and/or lack of transportation. The experimenter completed the experiment at the participant’s home for 3 of 16 individuals with aphasia in Experiment 1.

### 3.8 fNIRS data processing

FNIRS data were processed in Homer3 v1.87.0 (Huppert et al., 2009). When available for individuals with aphasia, pre-existing T1-weighted structural MRI scans were used, either from previous research participation or from clinical MRIs. For participants who had completed a recent study in the Center for Brain Recovery, T1-weighted structural MRI scans and corresponding lesion maps drawn using ITK-SNAP^2^ (Yushkevich et al., 2006) were available. For participants with research MRIs and lesion maps available, head measurements were input into AtlasViewer (Aasted et al., 2015) and estimated individual MNI coordinates for the center of each channel were generated. Following this, channel center coordinates falling within lesioned tissue were identified by viewing the T1 and lesion map in MRIcron. Subsequently, channels identified using MRIcron were manually excluded at the participant level in Homer3 (Huppert et al., 2009) prior to running the data processing stream to minimize any potential contribution of spurious signal from the area of lesion (Gilmore et al., 2021).

For participants with clinical MRIs, the scans were reviewed, general lesion location was confirmed, and channels were omitted from processing manually. For participants without research or clinical MRIs, all left-hemisphere channels were excluded. For Experiment 1, 11 participants had research scans and lesion maps available; 3 participants had clinical MRIs available; and 2 participants did not have MRI data available (see Table 4 for list of fNIRS channels omitted by participant in Experiment 1).

**Table 4.** Experiment 1 number of participants with data for each ROI after elimination of lesioned channels (PWA) and after processing stream channel pruning (all groups).

	Left hemisphere				Right hemisphere			
Group	Frontal	Precentral	Temporal	Parietal	Frontal	Precentral	Temporal	Parietal
Young (/15)	15	15	15	15	15	15	15	15
Age-matched (/15)	15	15	15	15	15	15	15	15
PWA (/16)	11	11	9	10	16	16	16	16

In addition to exclusion of channels in lesioned tissue, additional time with significant artifacts (e.g., if fNIRS collection was not stopped promptly at the end of a run) was manually excluded. In addition, channels determined to be collecting data from lesioned tissue were manually excluded at the participant level. Then, the following processing stream was completed: (1) pruning channels at the run level within each participant with signal-to-noise ratio < 5 dB; (2) conversion of raw light intensity data to optical density; (3) motion artifact correction using a spline interpolation method with Savitsky–Golay filtering (frame size = 10 seconds; *p* = .99; Jahani et al., 2018); (4) low-pass filtering to remove high-frequency noise unrelated to the neural signal (low-pass filter frequency = .5 Hz); (5) conversion of optical density to concentration units using the modified Beer–Lambert Law; and (6) estimation of a generalized linear model (GLM) at the run level for changes in HbO and HbR over the time course of each block with short separation regression (performed with the short separation channel with the greatest correlation to each channel) and signal drift correction (trange = -2–55; see Supplementary Material for Experiment 1 processing stream parameters). After running the processing stream, optical density and post-GLM processed data were reviewed visually across channels at the run, participant, and group level. Channels that were highly aberrant at the run level (i.e., substantially greater periodicity or amplitude in post-processed data than other channels in that run) were manually excluded as well as stimulus markers with highly aberrant data across channels.^3^ The processing stream was then re-run.

### 3.9 Statistical analysis

For each participant, channel-level data were averaged across channels into eight broad ROIs: bilateral frontal (IFG and MFG), bilateral PCG, bilateral temporal (MTG and STG), and bilateral parietal lobe (AG and SMG).

Across models, a random intercept for participant was included to account for individual differences. In cases where a model did not converge, the random intercept was removed. Condition was dummy coded with repeat as the reference variable and group (when applicable) was dummy coded with age-matched (i.e., older healthy) participants as the reference variable. Talk time (i.e., average time in a trial when the participant stopped talking) was used as a covariate of no interest to account for variable talk times and potential variable physiological noise across conditions, groups, and participants. Choral cues were not incorporated into statistical models given the small sample size. Given the preliminary nature of these experiments, both corrected and uncorrected results are reported.

#### 3.9.1 Research Question 1

To account for variation in HbO over the time course of the block, for each group separately, HbO was averaged into three epochs (0–16.5 seconds, 16.5–33 seconds, and 33–48.5 seconds), corresponding to roughly 1/3 of the block for each epoch. Then, two groups of mixed effects models were built for each ROI in each group. In the first group of models, predictors were the interaction between condition (questions vs. repeat) and epoch (1, 2, or 3), with epoch treated as a factor where Epoch 1 was the reference level. This model group is reported in the Supplementary Material. In the second group of models, predictors were condition and epoch. FDR correction (Benjamini & Hochberg, 1995) was completed to account for multiple comparisons using the p.adjust function in R.

#### 3.9.2 Research Question 2

Estimated HbO values over the time course of 10–48.5 seconds in the block were averaged within conditions and within groups. Ten seconds was chosen to allow initial rise time of the neural signal and 48.5 seconds was chosen as this was the end of a block. Then, two groups of models were built. In the first group, a separate mixed effects model was built for each of the eight ROIs using group, condition, and the interaction between group and condition to predict HbO. These results are reported in the Supplementary Material. In the second group of models, predictors were group and condition. FDR correction (Benjamini & Hochberg, 1995) was completed to account for multiple comparisons using the p.adjust function in R.

For both research questions, analysis was repeated using HbR given the recommendation to include both chromophores in fNIRS analysis (Yücel et al., 2021). Results for HbR are reported in the Supplementary Material.

## 4 Experiment 1 Results

Average Western Aphasia Battery – Revised Aphasia Quotient was 83 (SD = 16.2, range = 54.1–100), indicative of mild aphasia severity on average with a range from moderate to minimal. Average Cognitive Linguistic Quick Test Plus composite score was 3.5 (SD = 0.5, range = 2.4 4), indicative of mild severity of non-linguistic cognitive impairment on average with a range from moderate to within normal limits (see Table 5 for behavioral testing results for people with aphasia).

**Table 5.** Experiment 1 test scores for people with aphasia.

ID	WAB-R AQ	Aphasia Subtype per WAB-R	WAB-R repetition	WAB-R picture description global coherence	WAB-R picture description type token ratio	ASRS v3.5 score	CLQT+ composite score
A1	89.9	Anomic	93	3.5	0.51	0	3.8
A2	97.8	Not aphasic*	100	3.8	0.31	6	4
A3	90	Anomic	81	2.9	0.50	2	3.2
A4	95.3	Not aphasic*	90	3.9	0.55	2	3.6
A5	72.5	Conduction	60	NA	NA	3	3.6
A6	54.1	Broca’s	41	1.3	0.59	25	2.4
A7	91.4	Anomic	88	4.0	0.51	1	3.8
A8	93.2	Anomic	85	3.9	0.48	2	4
A9	96.4	Not aphasic*	97	3.9	0.34	6	4
A10	55.8	Broca’s	40	NA	NA	23	2.8
A11	96	Anomic	98	3.7	0.69	2	3.4
A12	94.9	Not aphasic*	98	3.9	0.55	5	3.2
A13	72.1	Wernicke’s	73	3.8	0.68	2	3.2
A14	100	Not aphasic*	100	4.0	0.53	0	4
A15	66.3	Conduction	52	3.0	0.48	12	3.2
A17	61.7	Conduction	37	2.6	0.50	6	3.2
Mean	83		77.1	3.4	0.51	6.1	3.5
SD	16.2		23.3	0.8	0.10	7.6	0.5

WAB-R AQ – Western Aphasia Battery Revised – Aphasia Quotient (scores range from 0 to 100 with higher scores indicative of more mild aphasia); *Not aphasic by WAB-R AQ cutoff, although still presenting with minimal aphasia per clinical judgment; Global coherence scores range from 1 (unrelated utterance) to 4 (overtly related to the topic); ASRS v3.5 – Apraxia of Speech Rating Scale version 3.5 (scores range from 0 to 52 with higher scores representing motor speech impairment); CLQT+ – Cognitive Linguistic Quick Test Plus Composite Scores (scores range from 1 to 4 with higher scores indicative of more mild impairment).

See Supplementary Material for Experiment 1 statistical model output.

### 4.1 Research Question 1: In three groups (young neurotypical individuals, individuals with post-stroke aphasia, and age-matched neurotypical individuals), can fNIRS be used to index cortical activity differences between language formulation (i.e., conversational responses) and sentence repetition?

**Fig. 3.**
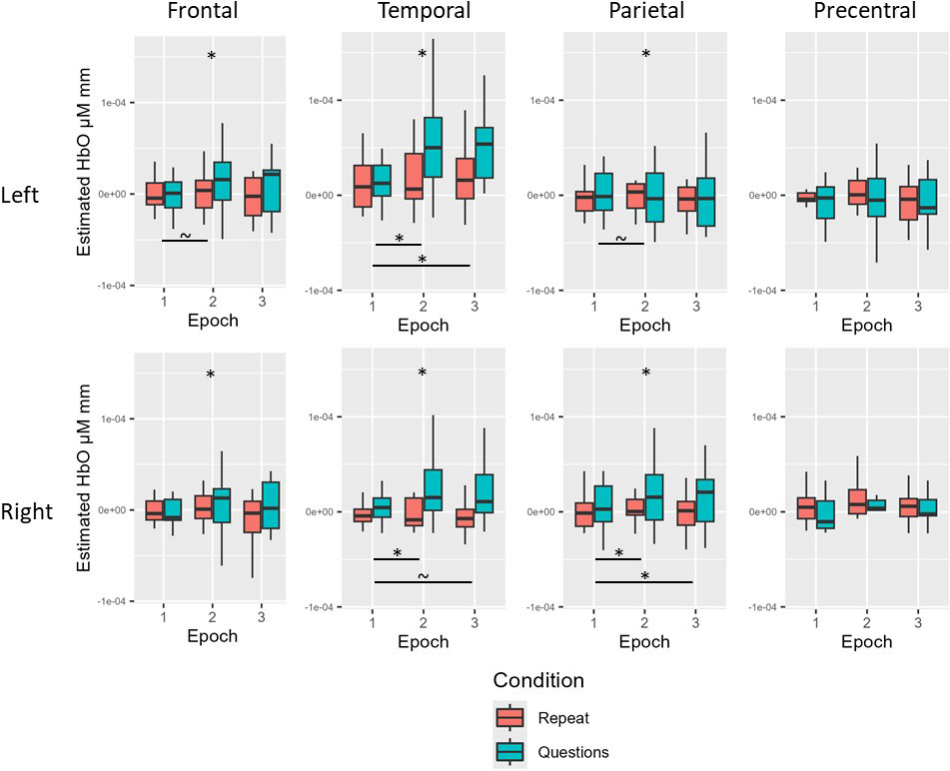
Experiment 1 young neurotypical results. Before multiple comparison correction (~), there was a simple effect of epoch for HbO in the left frontal (Epoch 2 > Epoch 1), left parietal (Epoch 2 > Epoch 1), and right parietal (Epoch 3 > Epoch 1) ROIs. After multiple comparison correction (*), there remained a simple effect of condition for HbO in the left frontal, right frontal, left temporal, right temporal, left parietal, and right parietal ROIs (questions > repeat). There also remained a simple effect of epoch for HbO in the left temporal (Epoch 2 > Epoch 1; Epoch 3 > Epoch 1), right temporal (Epoch 2 > Epoch 1), and right parietal (Epoch 2 > Epoch 1; Epoch 3 > Epoch 1) ROIs.

For the young neurotypical group before multiple comparison correction, there was a simple effect of condition for HbO in the bilateral frontal, temporal, and parietal ROIs (questions > repeat; all *p* < .05). There was also a simple effect of epoch for HbO in the left frontal (Epoch 2 > Epoch 1), left temporal (Epoch 2 > Epoch 1; Epoch 3 > Epoch 1), right temporal (Epoch 2 > Epoch 1; Epoch 3 > Epoch 1), left parietal (Epoch 2 > Epoch 1), and right parietal (Epoch 2 > Epoch 1; Epoch 3 > Epoch 1) ROIs (all *p* < .05). After multiple comparison correction, there remained a simple effect of condition for HbO in the bilateral frontal, temporal, and parietal ROIs (questions > repeat, all adjusted *p* < .05). There also remained a simple effect of epoch for HbO in the left temporal (Epoch 2 > Epoch 1; Epoch 3 > Epoch 1), right temporal (Epoch 2 > Epoch 1), and right parietal (Epoch 2 > Epoch 1; Epoch 3 > Epoch 1) ROIs (all adjusted *p* < .05; see Table 6 and Fig. 3 for Experiment 1 young neurotypical results).

**Table 6.** Summary of Experiment 1 results.

**Left**				
Group	Frontal	Temporal	Parietal	Precentral
Young	Questions > Repeat* *d* *=* *.3* Epoch 2 > Epoch 1 *d* *=* *.4*	Questions > Repeat* *d* *=* *.64* Epoch 2 > Epoch 1* *d* *=* *.36* Epoch 3 > Epoch 1* *d* *=* *.4*	Questions > Repeat* *d* *=* *.11* Epoch 2 > Epoch 1 *d* *=* *.2*	
Age-Matched			Questions > Repeat *d* *=* *.55* Epoch 2 > Epoch 1 *d* *=* *.38* Epoch 3 > Epoch 1 *d* *=* *.42*	
People with Aphasia			Epoch 2 > Epoch 1 *d* *=* *.48*	
Combined		Questions > Repeat* *d* *=* *.5* Young > Age-matched* *d* *=* *.95*	Questions > Repeat *d* *=* *.23*	

**Right**				
Group	Frontal	Temporal	Parietal	Precentral
Young	Questions > Repeat* *d* *=* *.41*	Questions > Repeat* *d* *=* *.66* Epoch 2 > Epoch 1* *d* *=* *.47* Epoch 3 > Epoch 1 *d* *=* *.38*	Questions > Repeat* *d* *=* *.35* Epoch 2 > Epoch 1* *d* *=* *.46* Epoch 3 > Epoch 1* *d* *=* *.39*	
Age-Matched	Epoch 3 > Epoch 1 *d* *=* *.42*		Epoch 3 > Epoch 1 *d* *=* *.55*	
People with Aphasia				
Combined	PWA > Age-matched *d* *=* *.82*		Young > Age-matched *d* *=* *.5* PWA > Age-matched *d* *=* *.69*	Young > Age-matched *d* *=* *.4* PWA > Age-matched* *d* *=* *1.02*

* Results remained significant after multiple comparison correction.

d = Cohen’s d (measure of effect size).

Repeat was the reference level for Condition and Epoch 1 was the reference level for Epoch. In the combined data (Research Question 2), age-matched was the reference level for group.

**Fig. 4.**
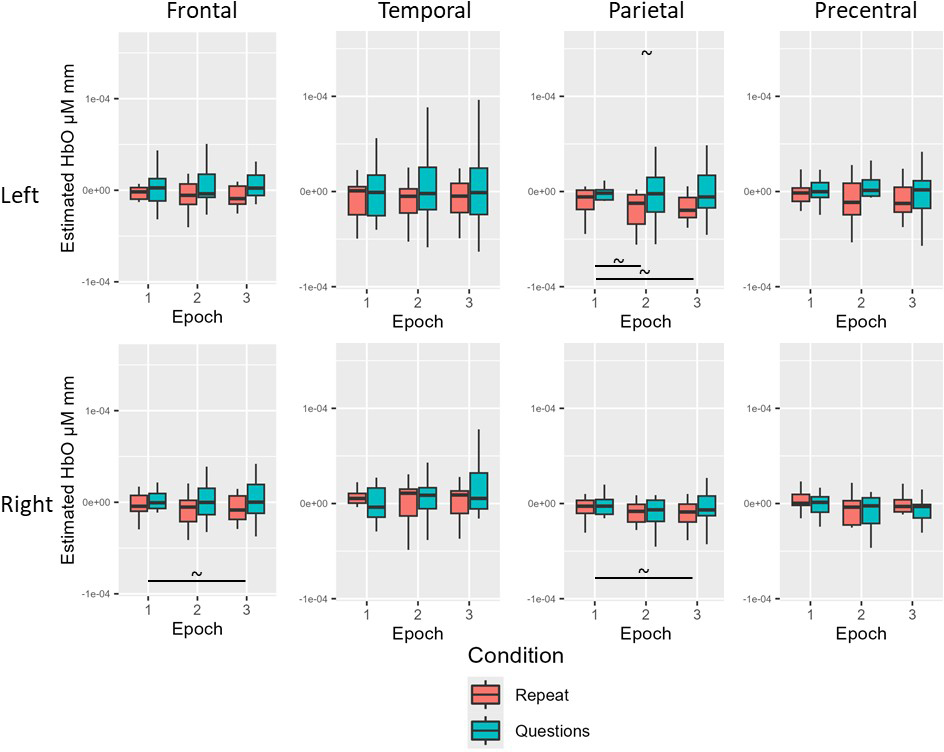
Experiment 1 age-matched neurotypical results. Before multiple comparison correction (~), there was a simple effect of condition for HbO in the left parietal ROI (questions > repeat) and a simple effect of epoch for HbO in the right frontal (Epoch 3 > Epoch 1), left parietal (Epoch 2 > Epoch 1; Epoch 3 > Epoch 1), and right parietal (Epoch 3 > Epoch 1) ROIs. After multiple comparison correction, there were no significant effects of condition or epoch for HbO across ROIs.

For the age-matched neurotypical group before multiple comparison correction, there was a simple effect of condition for HbO in the left parietal ROI (questions > repeat) and a simple effect of epoch for HbO in the right frontal (Epoch 3 > Epoch 1), left parietal (Epoch 2 > Epoch 1; Epoch 3 > Epoch 1), and right parietal (Epoch 3 > Epoch 1) ROIs (all *p* < .05). For the age-matched neurotypical group after multiple comparison correction, there were no significant effects of condition or epoch for HbO across ROIs (all adjusted *p* > .05; see Table 6 and Fig. 4 for Experiment 1 age-matched results).

**Fig. 5.**
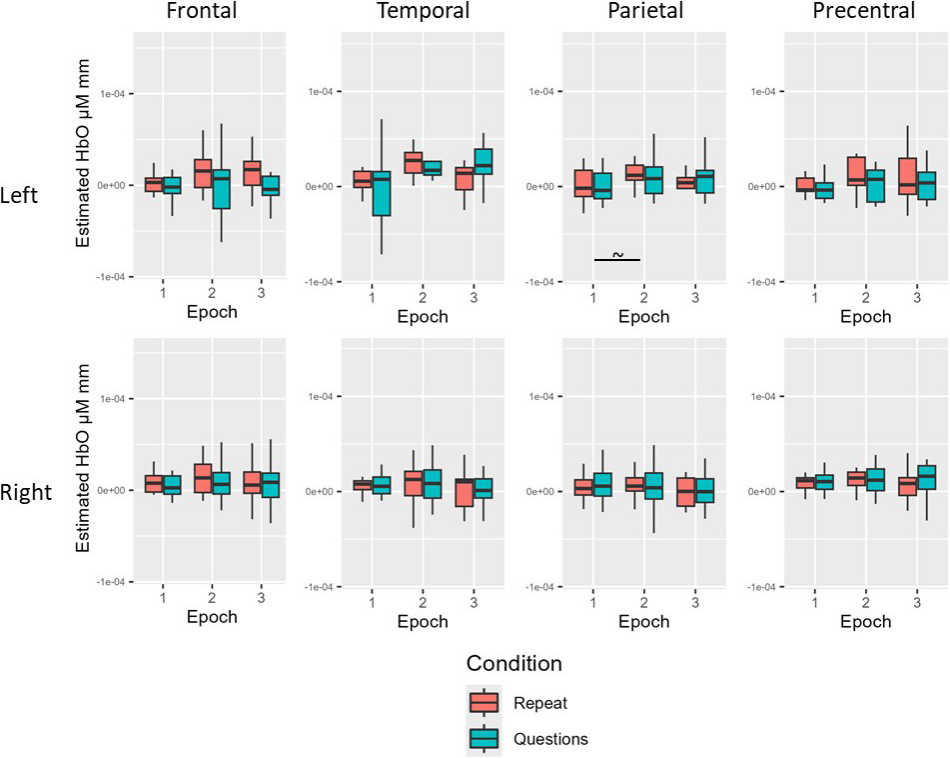
Experiment 1 individuals with aphasia results. Before multiple comparison correction (~), there was a simple effect of epoch for HbO in the left parietal ROI (Epoch 2 > Epoch 1). After multiple comparison correction, there were no significant effects of condition or epoch for HbO across ROIs.

For the individuals with aphasia before multiple comparison correction, there was a simple effect of epoch for HbO in the left parietal ROI (Epoch 2 > Epoch 1; *p* = .036). For the individuals with aphasia after multiple comparison correction, there were no significant effects of condition or epoch for HbO across ROIs (all adjusted *p* > .05; See Table 6 and Fig. 5 for Experiment 1 individuals with aphasia results).

### 4.2 Research Question 2: Are there differences in cortical activity for conversational responses and sentence repetition among young neurotypical individuals, individuals with post-stroke aphasia, and age-matched neurotypical individuals?

**Fig. 6.**
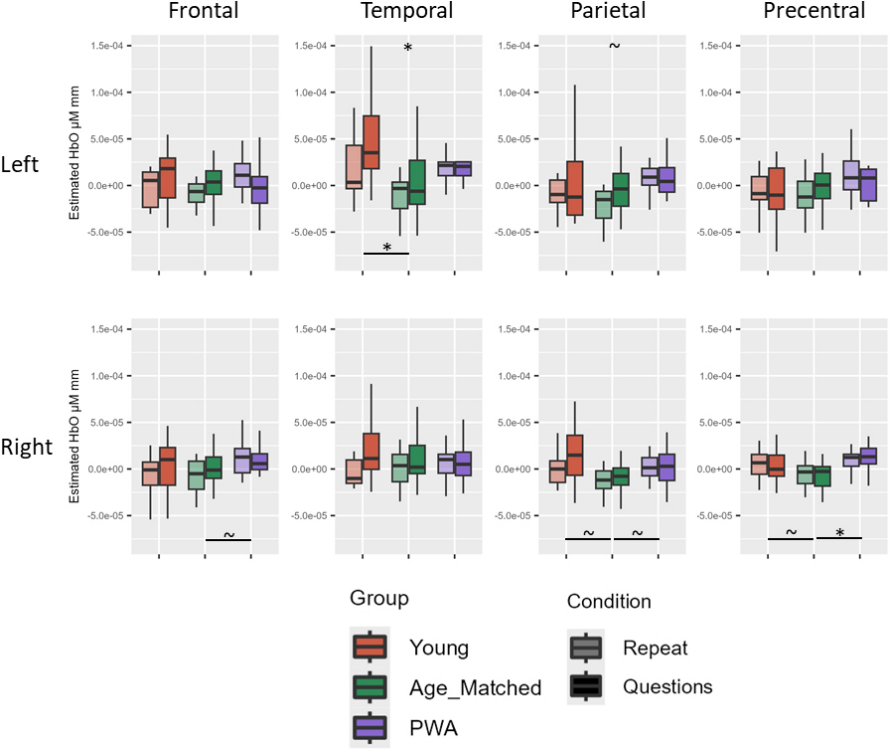
Experiment 1 group comparison results. Before multiple comparison correction (~), there was a simple effect of condition in the left parietal ROI (questions > repeat). Before multiple comparison correction, there was also a simple effect of group for HbO in the right frontal (PWA > age-matched), right parietal (young > age-matched; PWA > age-matched), and right PCG (young > age-matched). After multiple comparison correction (*), there remained a simple effect of condition for HbO in the left temporal ROI (questions > repeat) and a simple effect group for HbO in the left temporal (young > age-matched) and right PCG (PWA > age-matched).

Before multiple comparison correction, there was a simple effect of condition for HbO in the left temporal and left parietal ROIs (questions > repeat; all *p* < .05). There was also a simple effect of group for HbO in the right frontal (PWA > age-matched), left temporal (young > age-matched), right parietal (young > age-matched; PWA > age-matched), and right PCG (young > age-matched; PWA > age-matched) ROIs (all *p* < .05). After multiple comparison correction, there remained a simple effect of condition for HbO in the left temporal ROI (questions > repeat) and a simple effect of group for HbO in the left temporal (young > age-matched) and right PCG (PWA > age-matched) ROIs (all adjusted *p* < .05; see Table 6 and Fig. 6 for averaged data across all groups for Experiment 1).

## 5 Interim Comments

In Experiment 1, because of the goal to emulate turn-taking seen in naturalistic conversation, the task was designed with blocks of three consecutive questions. However, the resultant data show three distinct regions of rise and fall in HbO. For this reason, Experiment 2 was modified to include one-question blocks rather than three-question blocks. In addition, Experiment 1 was designed as a computer-based conversation task, but the goal of this line of work is to be able to evaluate cortical activity in functional communication tasks. Toward this goal, Experiment 2 modified Experiment 1 with the primary difference being that the conversational questions were administered by a live interlocutor rather than by video-recorded clips presented on a computer.

## 6 Experiment 2 Methods

### 6.1 Participants

Recruitment strategy and eligibility criteria were the same in Experiment 2 as Experiment 1. In Experiment 2, participants were 15 young neurotypical adults (mean age = 21.8 years, SD = 3.1; 11 female, 4 male), 16 individuals with chronic (i.e., greater than 6 months) post-stroke aphasia (mean age = 58.9 years, SD = 9.5; 5 female, 11 male), and 15 age-matched neurotypical adults (mean age = 64 years, SD = 9.6; 9 female, 6 male; see Table 7 for young and age-matched demographics and Table 8 for individuals with aphasia demographics). Five young participants reported a history of depression. One age-matched participant reported a history of depression and one reported a history of mild concussion. For the hearing screening, 13 of 15 age-matched participants passed in at least one ear and 10 of 16 people with aphasia passed in at least one ear. All age-matched neurotypical participants scored within normal limits on the Mini Mental State Examination, 2nd Edition Expanded Version (Folstein & Folstein, 2010) as a cognitive screening (average t-score = 56, standard deviation = 5, range = [46, 63]).^4^ One young neurotypical individual, 11 age-matched neurotypical individuals, and 11 individuals with aphasia also participated in Experiment 1.

**Table 7.** Experiment 2 young and age-matched neurotypical demographics.

ID	Age	Sex	Handedness	Race	Ethnicity	Native language	Other languages	Years educ.
Y1	23	M	L	White	H	Spanish	English, Latin, French, Portuguese	16
Y2^	22	M	R	White	H	English	Spanish, Danish, Japanese	16
Y3	19	M	R	White	NH	English	Mandarin	13
Y4	19	M	R	Asian, White	NH	English	Italian*	13
Y5	20	F	R	White	NH	English	-	14
Y6	21	F	L	Asian	NH	English	Spanish*, Mandarin*	16
Y7	22	F	R	White	NH	English	-	15
Y8	20	F	R	White	NH	English	-	13
Y9	22	F	R	White	H	Spanish, English	-	15
Y10	30	F	R	White	NH	English	Spanish*	16
Y11	21	F	R	Asian	NH	English, Urdu	-	14
Y12	27	F	R	Asian	NH	English	Korean	16
Y13	20	F	R	White	NH	English	Dutch, French	17
Y14	19	F	R	Asian, White	NH	English	Spanish*, Cantonese*	13
Y15	22	F	R	Black	NH	English	-	16
AM1^	65	F	R	White	NH	English	-	17
AM2^	52	M	R	White	NH	Portuguese	English, Spanish	12
AM3^	60	M	R	White	NH	English	-	16
AM4^	63	F	R	White	NH	English	French*	16
AM5^	59	M	R	White	NH	English	-	16
AM6^	46	M	R	White	NH	English	Spanish, French, Portuguese, Arabic	16
AM7^	51	M	R	White	NH	English	-	16
AM8^	69	F	R	White	NH	English	-	18
AM9^	80	F	R	White	NH	English	-	19
AM10	69	M	R	White	NH	English	-	32
AM11^	64	F	R	White	NH	English	Spanish, French	17
AM12^	72	F	R	White	NH	English	-	16
AM13	70	F	R	White	NH	English	German*	20
AM14	74	F	R	White	NH	English	Hebrew, French	21
AM15	66	F	R	White	NH	English	-	17

^ Also participated in Experiment 1.

* Basic or intermediate.

Y: young; AM: age-matched; NH: non-Hispanic; H: Hispanic; Educ: education; M: male; F: female.

**Table 8.** Experiment 2 demographics for people with aphasia.

ID	Age	Sex	Hand.	Race	Ethnicity	Native language	Other languages	Years educ.	MPO	Stroke location (per medical record)
A1^	69	M	R	White	NH	English	None	16	62	Left MCA
A2^	69	M	L	White	NH	English	None	17	78	Left MCA (fronto-temporoparietal)
A3^	58	F	R	Black	NH	English	None	16	77	Left hemisphere*
A4^	60	M	L	White	NH	English	None	12	303	Left hemisphere
A5	55	F	R	Black	NH	English	None	14	53	N/A
A6^	60	F	R	White	NH	English	Spanish	17	158	Left hemisphere (frontoparietal)
A7^	58	M	R	White	NH	English	None	16	54	Left MCA
A8^	57	M	R	White	NH	English	None	16	21	Left hemisphere (thalamus, basal ganglia)
A9^	36	F	R	White	NH	English	Spanish	18	12	Left hemisphere
A10	57	M	R	White	NH	English	None	20	38	Left hemisphere (inferior frontal, insula)
A11	56	F	R	White	NH	English	None	18	90	Left PCA and MCA
A12^	58	M	R	Black	NH	English	None	12	159	Left MCA (frontotemporal, subcortical)
A13^	63	M	R	White	NH	English	Spanish	19	29	Left MCA (parietal)
A14	45	M	R	White	NH	English	None	12	11	Left hemisphere
A15^	76	M	R	White	NH	English	None	20	92	Left MCA
A16	65	M	R	White	NH	English	None	18	47	Left hemisphere
Mean	58.9							16.3	80.3	
SD	9.3							2.7	74.1	

^ Also participated in Experiment 1.

* History of multiple strokes.

M – male; F – female; Hand – handedness; R – right; L – left; H – Hispanic; NH – non-Hispanic; MPO – months post-onset.

### 6.2 Behavioral assessment

Behavioral assessment for Experiment 2 was the same as behavioral assessment for Experiment 1.

### 6.3 Experimental task development

The Experiment 1 task was modified for Experiment 2 based on data collection and observations from that experiment. Given that separate events were observable in the multi-question block from Experiment 1 time course data, Experiment 2 was modified to include only one question and answer per block. In addition, questions were a subset of those from Experiment 1, with the following question types eliminated: questions that elicited below- or above-average response length; questions that contained an above-average number of words; and questions that were overly culturally specific (see Supplementary Material for list of questions). Last, the response period was increased from 10 to 15 seconds given that neurotypical participants were intermittently unable to complete their full response in the 10-second period in Experiment 1. Behavioral task programming in Experiment 2 was similar to that in Experiment 1.

### 6.4 Experimental task design

Experiment 2 comprised four runs of approximately 6 minutes each (compared with 7-minute runs in Experiment 1). Each run contained six question and answer blocks (experimental condition) comprising a question period (5 seconds) and an answer period (15 seconds) and four repeat blocks (control condition) comprising a sentence listening period (5 seconds) and a repetition period (15 seconds). In contrast to Experiment 1, the experimenter and participant were seated across from each other with a small screen in between them and questions were presented by the experimenter. During the question and answer block, the screen was blank and the experimenter asked the participant the scripted open-ended question. The participant was instructed to answer during the 15-second response period. During the repeat blocks, the screen was blank and the experimenter stated a sentence (*The dog is barking and the cat is sleeping*). The participant was instructed to repeat the sentence aloud three times during the response period. As in Experiment 1, during the response period, participants saw a progress bar on the screen in order for them to gauge time remaining to answer each question.

**Fig. 7.**
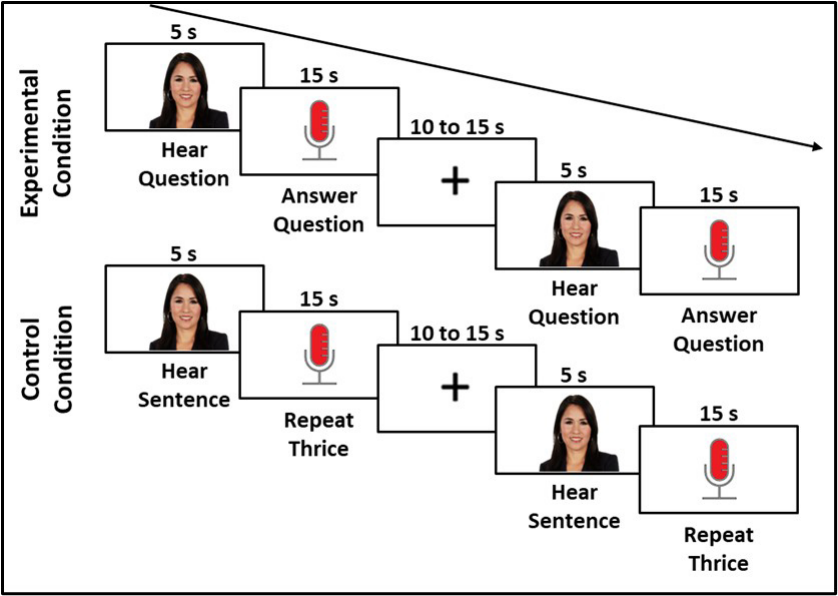
Experiment 2 task design.

**Appendix Fig. A3:**
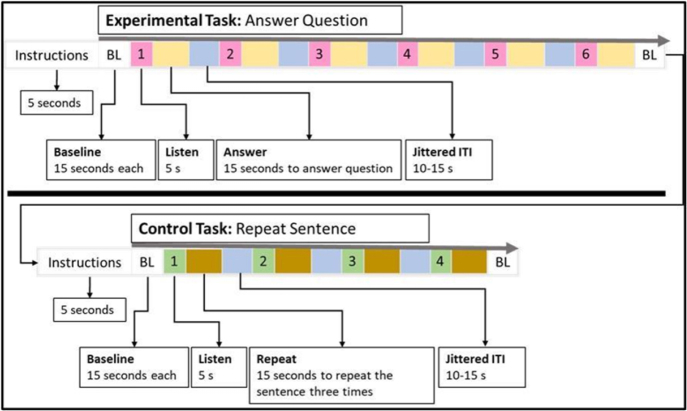
Experiment 2 task timing.

Other randomization, counterbalance, and timing features of Experiment 2 were the same as Experiment 1 with the exception that the inter-stimulus interval between blocks varied randomly between 10 and 15 seconds (see Fig. 7 for Experiment 2 task design and Appendix Fig. A3 for Experiment 2 task timing).

As in Experiment 1, participants completed a practice task after receiving verbal instructions about the task. The practice task included three question and answer blocks and two repeat blocks. Participants were then given an opportunity to ask questions about task completion and allowed to repeat the practice task if necessary for comfort with the task demand.

Task modifications for people with aphasia were the same as Experiment 1 with two exceptions. First, for one participant, the questions were read aloud prior to each run to reduce formulation demands. Second, if participants were unable to repeat the sentence three times during a response period in the practice task, they were instructed to only repeat the sentence twice (N = 4).

### 6.5 Evaluation of task performance

Evaluation of task performance in Experiment 2 mirrored evaluation of task performance in Experiment 1. See Table 9 for talk time average values across groups and conditions for Experiment 2.

**Table 9.** Average end of talking time per trial Experiment 2.

Group	Questions	Repeat
Young	10.4 (1.5)	8.4 (1.1)
Age-matched	12.1 (1.5)	9.3 (1.3)
People with Aphasia	11.7 (1.7)	11 (1.1)
Overall	11.4 (0.9)	9.8 (0.3)

Mean (standard deviation) in seconds (each trial is 15 seconds in length).

### 6.6 fNIRS probe design

Experiment 2 used the same fNIRS probe as in Experiment 1.

### 6.7 fNIRS data collection

Data collection followed the same set-up as in Experiment 1. The experiment was completed at the participant’s home for 5 of 16 individuals with aphasia in Experiment 2.

### 6.8 fNIRS data processing

FNIRS data pre-processing steps were the same as in Experiment 1 with the exception of the time range for the GLM (trange = -2–25 seconds; see Supplementary Material for Experiment 2 processing stream parameters).

Evaluation of structural MRIs was the same in Experiment 2 as in Experiment 1. In Experiment 2, nine participants had research scans and lesion maps available; two participants had clinical MRIs available; and five participants did not have MRI data available (see Table 10 for fNIRS channels omitted in Experiment 2).

**Table 10.** Experient 2 number of participants with data for each ROI after elimination of lesioned channels (PWA) and after processing stream channel pruning (all groups).

	Left hemisphere				Right hemisphere			
Group	Frontal	Precentral	Temporal	Parietal	Frontal	Precentral	Temporal	Parietal
Young (/15)	15	14	14	15	15	14	15	15
Age-matched (/15)	15	15	15	15	15	15	15	15
PWA (/16)	11	11	9	10	16	16	16	16

### 6.9 Statistical analysis

#### 6.9.1 Research Question 1

To account for variation in HbO over the time course of the block, for each group separately, HbO was averaged into four epochs (0–5 seconds, 5–10 seconds, 10–15 seconds, and 15–20 seconds), corresponding to 1/4 of the block for each epoch. Then, in one group of models, separate mixed effects models were built for each ROI in each group, with predictors being condition (questions vs. repeat), epoch (1, 2, 3, or 4), and the interaction between condition and epoch, with epoch treated as a factor where Epoch 1 was the reference level. These results are reported in the Supplementary Material. In a second group of models, predictors were condition and epoch. FDR correction (Benjamini & Hochberg, 1995) was completed to account for multiple comparisons using the p.adjust function in R.

#### 6.9.2 Research Question 2

Estimated HbO values over the time course of 5–15 seconds in the block were averaged within conditions and within groups. As in Experiment 1, separate mixed effects models were built for each of the eight ROIs using group, condition, and the interaction between group and condition to predict HbO. These results are reported in the Supplementary Material. For the second group of models, predictors were group and condition without the interaction term. FDR correction (Benjamini & Hochberg, 1995) was completed to account for multiple comparisons using the p.adjust function in R.

For both research questions, analysis was repeated using HbR and these results are reported in the Supplementary Material.

## 7 Experiment 2 Results

Average Western Aphasia Battery – Revised Aphasia Quotient was 84.4 (SD = 17.7, range = 49.7–100), indicative of mild aphasia severity on average with a range from moderate to minimal. Average Cognitive Linguistic Quick Test Plus non-linguistic cognitive score was 3.5 (SD = 0.5, range = 2.4–4), indicative of mild severity of non-linguistic cognitive impairment on average with a range from moderate to within normal limits (see Table 11 for Experiment 2 behavioral data for people with aphasia).

**Table 11.** Experiment 2 test scores for people with aphasia.

ID	WAB-R AQ	WAB-R Repetition	Aphasia Subtype per WAB-R	WAB-R Picture Description Global Coherence	WAB-R Picture DescriptionType Token Ratio	ASRS v3.5 Score	CLQT+ Composite Score
A1^	93.2	85	Anomic	3.9	0.48	2	4
A2^	89.9	93	Anomic	3.5	0.51	0	3.8
A3^	96	98	Anomic	3.7	0.69	2	3.4
A4^	55.8	40	Broca’s	NA	NA	23	2.8
A5	99.6	98	Not aphasic*	4	0.525	1	3.8
A6^	96.4	97	Not aphasic*	3.9	0.34	6	4
A7^	61.7	37	Conduction	2.6	0.50	6	3.2
A8^	94.9	98	Not aphasic*	3.9	0.55	5	3.2
A9^	100	100	Not aphasic*	4.0	0.53	0	4
A10	98.1	94	Not aphasic*	3.28	0.418	2	4
A11	95.2	92	Not aphasic*	3.47	0.469	1	3.8
A12^	90	81	Anomic	2.9	0.50	2	3.2
A13^	97.8	100	Not aphasic*	3.8	0.31	6	4
A14	61.6	64	Conduction	3.11	0.633	30	3.2
A15^	49.7	46	Broca’s	NA	NA	25	2.4
A16	71.2	53	Broca’s	1.9	0.526	NA	2.8
Mean	84.4	79.8		3.4	0.50	7.4	3.5
SD	17.7	23.4		0.6	0.10	9.9	0.5

WAB-R AQ – Western Aphasia Battery Revised – Aphasia Quotient (scores range from 0 to 100 with higher scores indicative of more mild aphasia); ^also participated in Experiment 1. *Not aphasic by WAB-R AQ cutoff, although still presenting with minimal aphasia per clinical judgment; Global coherence scores range from 1 (unrelated utterance) to 4 (overtly related to the topic); ASRS v3.5 – Apraxia of Speech Rating Scale version 3.5 (scores range from 0 to 52 with higher scores representing motor speech impairment); CLQT+ – Cognitive Linguistic Quick Test Plus Composite Scores (scores range from 1 to 4 with higher scores indicative of more mild impairment).

See Supplementary Material 6 for Experiment 2 statistical model output.

### 7.1 Research Question 1: In three groups (young neurotypical individuals, individuals with post-stroke aphasia, and age-matched neurotypical individuals), can fNIRS be used to index cortical activity differences between language formulation (i.e., conversational responses) and sentence repetition?

**Fig. 8.**
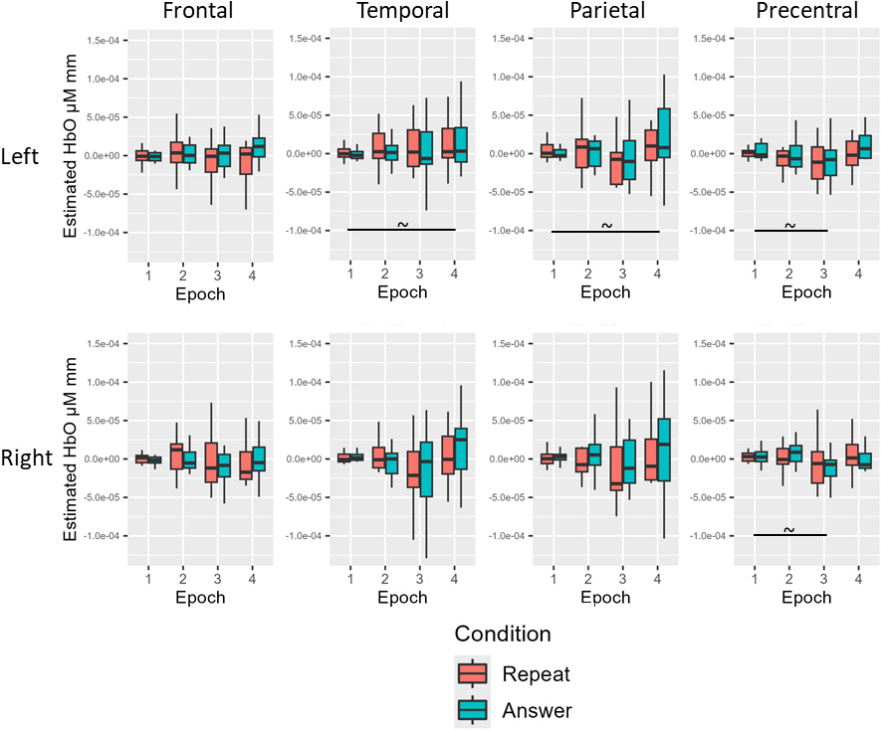
Experiment 2 young neurotypical results. Before multiple comparison correction (~), there was a simple effect of epoch for HbO in the left temporal (Epoch 4 > Epoch 1), left parietal (Epoch 4 > Epoch 1), left PCG (Epoch 3 < Epoch 1), and right PCG (Epoch 3 < Epoch 1). After multiple comparison correction, no significant HbO effects for condition or epoch.

For the young neurotypical group before multiple comparison correction, there was a simple effect of epoch for HbO in the left temporal (Epoch 4 > Epoch 1), left parietal (Epoch 4 > Epoch 1), left PCG (Epoch 3 < Epoch 1), and right PCG (Epoch 3 < Epoch 1; all *p* < .05). For the young neurotypical group after multiple comparison correction, there were no significant HbO effects for condition or epoch (all adjusted *p* > .05; see Table 12 and Fig. 8 for Experiment 2 young healthy results).

**Table 12.** Summary of results for Experiment 2.

Left				
Group	Frontal	Temporal	Parietal	Precentral
Young		Epoch 4 > Epoch 1 *d* *=* *.62*	Epoch 4 > Epoch 1 *d* *=* *.41*	Epoch 3 < Epoch 1 *d* *=* *-.57*
Age-Matched	Epoch 3 < Epoch 1 *d* *=* *-.61* Epoch 4 < Epoch 1* *d* *=* *-.89*	Epoch 4 < Epoch 1 *d* *=* *-.52*	Epoch 3 < Epoch 1* *d* *=* *-.92* Epoch 4 < Epoch 1* *d* *=* *-.85*	Epoch 3 < Epoch 1* *d* *=* *-.65* Epoch 4 < Epoch 1* *d* *=* *-.72*
People with Aphasia		Answer > Repeat *d* *=* *.42* Epoch 2 > Epoch 1 *d* *=* *.6* Epoch 3 > Epoch 1 *d* *=* *.58* Epoch 4 > Epoch 1 *d* *=* *.71*	Answer > Repeat *d* *=* *.06*	
Combined				

Right				
Group	Frontal	Temporal	Parietal	Precentral
Young				Epoch 3 < Epoch 1 *d* *=* *-.42*
Age-Matched	Epoch 3 < Epoch 1* *d* *=* *-.71* Epoch 4 < Epoch 1* *d* *=* *-1.13*		Epoch 3 < Epoch 1 *d* *=* *-.64* Epoch 4 < Epoch 1* *d* *=* *-.76*	Epoch 3 < Epoch 1* *d* *=* *-.52* Epoch 4 < Epoch 1* *d* *=* *-.63*
People with Aphasia	Epoch 2 > Epoch 1 *d* *=* *.42*	Answer > Repeat *d* *=* *.33*	Epoch 2 > Epoch 1 *d* *=* *.49*	
Combined				

* Results remained significant after multiple comparison correction.

d = Cohen’s d (measure of effect size).

Repeat was the reference level for Condition and Epoch 1 was the reference level for Epoch. In the combined data (Research Question 2), age-matched was the reference level for group.

**Fig. 9.**
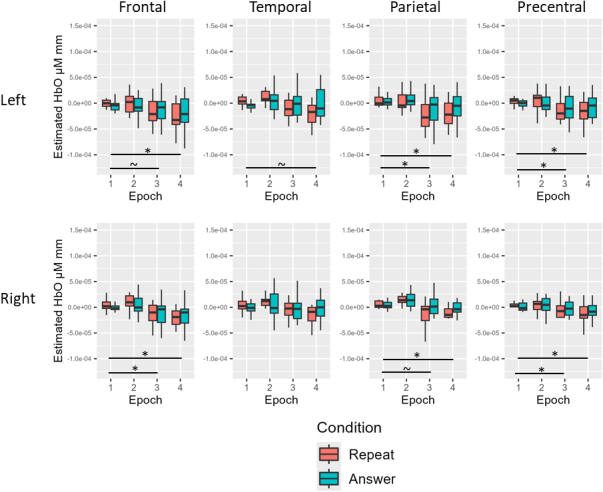
Experiment 2 age-matched neurotypical results. Before multiple comparison correction (~), there was a simple effect of Epoch for HbO in the left frontal (Epoch 3 < Epoch), left temporal (Epoch 4 < Epoch 1), and right parietal (Epoch 3 < Epoch 1). After multiple comparison correction (*), there remained a simple effect of epoch for HbO in the left frontal (Epoch 4 < Epoch 1), right frontal (Epoch 3 < Epoch 1; Epoch 4 < Epoch 1), left parietal (Epoch 3 < Epoch 1; Epoch 4 < Epoch 1), right parietal (Epoch 4 < Epoch 1), left PCG (Epoch 3 < Epoch 1; Epoch 4 < Epoch 1), and right PCG (Epoch 3 < Epoch 1; Epoch 4 < Epoch 1).

For the age-matched neurotypical group before multiple comparison correction, there was a simple effect of Epoch for HbO in the left frontal (Epoch 3 < Epoch 1; Epoch 4 < Epoch 1), right frontal (Epoch 3 < Epoch 1; Epoch 4 < Epoch 1), left temporal (Epoch 4 < Epoch 1), left parietal (Epoch 3 < Epoch 1; Epoch 4 < Epoch 1), right parietal (Epoch 3 < Epoch 1; Epoch 4 < Epoch 1), left PCG (Epoch 3 < Epoch 1; Epoch 4 < Epoch 1), and right PCG (Epoch 3 < Epoch 1; Epoch 4 < Epoch 1: all *p* < .05). For the age-matched neurotypical group after multiple comparison correction, there remained a simple effect of epoch for HbO in the left frontal (Epoch 4 < Epoch 1), right frontal (Epoch 3 < Epoch 1; Epoch 4 < Epoch 1), left parietal (Epoch 3 < Epoch 1; Epoch 4 < Epoch 1), right parietal (Epoch 4 < Epoch 1), left PCG (Epoch 3 < Epoch 1; Epoch 4 < Epoch 1), and right PCG (Epoch 3 < Epoch 1; Epoch 4 < Epoch 1; all adjusted *p* < .05; see Table 12 and Fig. 9 for Experiment 2 age-matched results).

**Fig. 10.**
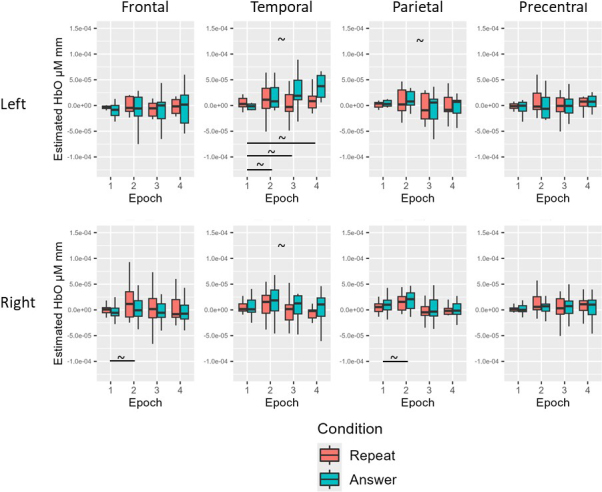
Experiment 2 individuals with aphasia results. Before multiple comparison correction (~), there was a simple effect of condition for HbO in the left temporal, right temporal, and left parietal ROIs (answer > repeat). There was also a simple effect of epoch for HbO in the right frontal (Epoch 2 > Epoch 1), left temporal (Epoch 2 > Epoch 1; Epoch 3 > Epoch 1; Epoch 4 > Epoch 1), and right parietal (Epoch 2 > Epoch 1) ROIs. After multiple comparison correction (*), there were no significant condition or epoch effects for HbO across ROIs.

For the people with aphasia before multiple comparison correction, there was a simple effect of condition for HbO in the left temporal, right temporal, and left parietal ROIs (answer > repeat; all *p* < .05). There was also a simple effect of epoch for HbO in the right frontal (Epoch 2 > Epoch 1), left temporal (Epoch 2 > Epoch 1; Epoch 3 > Epoch 1; Epoch 4 > Epoch 1), and right parietal (Epoch 2 > Epoch 1) ROIs (all *p* < .05). For the people with aphasia after multiple comparison correction, there were no significant condition or epoch effects for HbO across ROIs (all adjusted *p* > .05; see Table 12 and Fig. 10 for Experiment 2 individuals with aphasia results).

### 7.2 Research Question 2: Are there differences in cortical activity for conversational responses and sentence repetition among young neurotypical individuals, individuals with post-stroke aphasia, and age-matched neurotypical individuals?

**Fig. 11.**
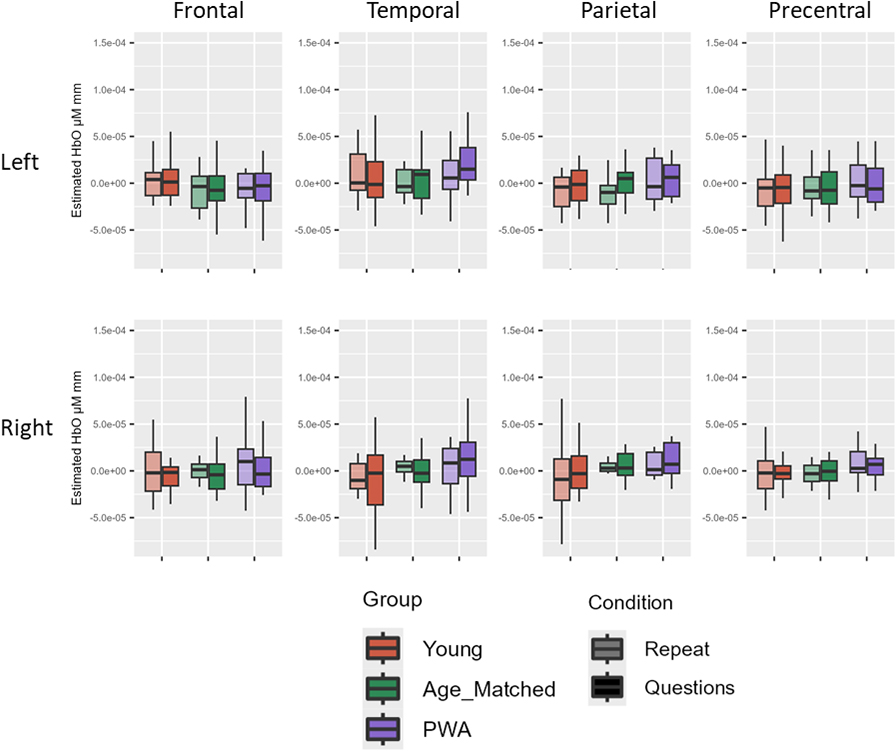
Experiment 2 group comparison results. There were no significant group or condition effects for HbO across ROIs before or after multiple comparison correction.

There were no significant group or condition effects for HbO across ROIs before or after multiple comparison correction (all *p* > .05; see Table 12 and Fig. 11 for averaged data across all groups for Experiment 2).

## 8 Discussion

This study evaluated cortical activity for conversational responses via fNIRS in young neurotypical individuals, individuals with post-stroke aphasia, and age-matched neurotypical individuals. Experiment 1 evaluated conversational responses in a highly structured computer-based conversation question task and showed (1) in the young neurotypical group, greater cortical activity for answering questions versus repeating in bilateral frontal, temporal, and parietal ROIs along with an overall increase in cortical activity across conditions over time in these ROIs; (2) in the age-matched group, greater cortical activity for answering questions versus repeating in the left parietal ROI along with an overall increase in cortical activity across conditions over time in the right frontal and bilateral parietal ROIs; (3) in the aphasia group, an overall increase in cortical activity in the left parietal ROI across conditions over time; (4) greater cortical activity in the left temporal ROI for young versus age-matched individuals; and (5) a pattern of greater cortical activity for the young group and people with aphasia compared with the age-matched group. When the experimental task was modified to involve a live conversational partner in Experiment 2, the following results emerged: (1) in the young neurotypical group, an overall increase in cortical activity in the left temporoparietal ROIs across conditions over time; (2) in the age-matched group, decreased cortical activity across conditions and ROIs bilaterally; and (3) in the aphasia group, greater cortical activity for answering questions versus repeating in bilateral temporal and left parietal ROIs along with an overall increase in cortical activity across ROIs over time in the left temporal, right frontal, and right parietal ROIs.

In Experiment 1, cortical responses for the young neurotypical group in the bilateral frontal, temporal, and parietal ROIs in the question condition were robust, differentiated from the repeat condition, and differentiated from individuals with aphasia and the age-matched (i.e., older) individuals. This result is consistent with the hypothesis and with the literature showing bilateral frontotemporal cortical responses for discourse tasks with formulation demands in healthy individuals (Suda et al., 2010). Results for the age-matched group and the individuals with aphasia were not consistent with the hypothesis that there would be differentiation between conditions in all three groups. Individuals with aphasia showing less cortical activity in the left hemisphere for language tasks than healthy controls is generally consistent with fMRI literature (Wilson & Schneck, 2021). However, given that this result was seen in both the age-matched and post-stroke aphasia groups, it may be the case that there is an age-related effect in cortical activity for this task compounded with a stroke-related effect.

Evaluating group differences directly in Experiment 1, there was greater cortical activity in the left temporal ROI for the young versus age-matched group. As above, this may be due to age-related cortical changes. In addition, there was greater cortical activity in the right PCG for people with aphasia versus the age-matched group. These results include talk time as a covariate, so it does not appear simply due to periods of speech output varying in length across groups. It may be the case that individuals with aphasia show greater right-hemisphere activity in compensation for their left-hemisphere stroke lesions. This is broadly consistent with previous findings showing increased cortical activity measured via fNIRS during picture naming in the right PCG for people with aphasia that was not seen in young and older control participants (Gilmore et al., 2021). This is also broadly consistent with the literature showing modest evidence for right-hemisphere recruitment for language tasks in individuals with post-stroke aphasia (Wilson & Schneck, 2021). Alternatively, this could reflect increased motor demand for the individuals with aphasia given that some presented with motor speech impairment.

In Experiment 2, while the task was more ecologically valid than that in Experiment 1, cortical responses were overall less robust than those in Experiment 1. For the young neurotypical group, in contrast to Experiment 1, there were no significant effects of condition (answer vs. repeat). One potential cause is experimental design differences. In Experiment 1, experimental blocks included three consecutive question and answer events. The single question plus answer block in Experiment 2 may not have had enough linguistic demand or overall cognitive load to elicit a clear cortical response via fNIRS for the young group.

For the age-matched group in Experiment 2, there was a pattern of decreased cortical activity across the course of a block. This is inconsistent with initial hypotheses. There are a few potential explanations for the absence of condition-related effects. First, it is possible that the age-matched group’s responses were generally reflective of low task demand similar to the young participants. In addition, it is possible that age-related changes led to overall decreased cortical activity across ROIs, although further investigation is needed to determine whether this result is anomalous.

For the people with aphasia in Experiment 2, before multiple comparison correction, there was significantly greater cortical activity in bilateral temporal and left parietal regions for the answer versus control condition. This result is consistent with the initial hypothesis and the literature in neurotypical individuals showing greater bilateral temporal activity for language formulation in conversational tasks versus speech production without formulation demands (Suda et al., 2010). This is broadly consistent with fMRI literature showing that people with aphasia tend to use left-hemisphere language regions for language tasks with potential recruitment of right-hemisphere homologues, but inconsistent with the fMRI literature showing generally reduced left-hemisphere cortical activity for language tasks compared with control participants (Wilson & Schneck, 2021). Another difference from Experiment 1 is the introduction of the live interlocuter in the task. The impact of the live interlocuter on performance and cortical activity requires further exploration in future studies.

Evaluating group differences directly in Experiment 2, there were no significant differences found between groups. This is not consistent with Experiment 1, again potentially due to task-related differences with three-question versus one-question blocks.

Overall, these results suggest engagement of bilateral frontal, temporal, and parietal cortical regions in healthy young speakers during conversational tasks involving receptive and expressive language. These findings suggest the engagement of a network of cortical regions for the complex task of understanding and producing phonological, lexical-semantic, and syntactic content at the level of discourse. Patterns varied between the young neurotypical group, age-matched neurotypical group, and individuals with aphasia, suggestive of changes in the neural mechanisms supporting conversational discourse production with both age and stroke-induced aphasia that can be detected by fNIRS. Furthermore, differences in results across tasks highlight the fact that varying discourse demands may involve different patterns of cortical involvement.

### 8.1 Limitations

There are some limitations to these experiments which should be mentioned. First, the task itself induced jaw-related motion artifacts which introduced noise in the signal and may have impacted the ability to detect a neural response. Several steps were taken in the data processing stream to ameliorate the effects of these artifacts and to remove channels and data time periods where significant artifacts could not be corrected. Future studies should consider cap designs to minimize the effect of jaw-related motion artifacts, including removal of the cap chin strap and attachment in other locations (e.g., around the back of the cap or secured on a chest strap).

In addition to jaw-related motion artifacts, speaking can induce systemic physiological changes, both intracerebral and extracerebral, that can be mistaken for task-based neural activity. Signals from short separation detectors were used as regressors to account for signal from the scalp unrelated to the neural signal. However, changes in systemic intracerebral physiology related to breathing could still have affected the results. Specifically, speaking induces changes in respiration which affects partial pressure of carbon dioxide in the arterial blood and ultimately the fNIRS signal (Tachtsidis & Scholkmann, 2016). To address this, a control condition was chosen that also involved speaking. However, average speaking times tended to be shorter in the control condition than in the experimental condition, so when there was a difference in the fNIRS signal between conditions that occurred late in a block, it is possible that this was due to differing systemic physiological changes based on the time that speaking ended. To further address this issue, average “end of talk time” by participant and by condition was used as a regressor in an attempt to account for this. Measurement and regression of additional physiological confounds, including respiration rate, in future work should help to ameliorate this concern (Tachtsidis & Scholkmann, 2016).

In addition to systemic physiological changes induced by speaking, there is also evidence of differing baseline physiology in older individuals that may affect study results (Amiri et al., 2014). For example, previous work has found that baseline cerebral blood flow (CBF) impacts the fMRI blood oxygenation level dependent (BOLD) response. If one group (e.g., older adults) tends to have lower baseline CBF, this could result in misleading results when comparing across different populations (Ances et al., 2009). This should be addressed in future work by controlling for baseline physiological differences between age groups.

For the individuals with aphasia, detailed lesion information was not available for all participants and so all left-hemisphere channels were excluded in those cases. This may have introduced bias into the experiment as this led to greater sample size for right-hemisphere ROIs. Ideally, future studies would have access to detailed lesion information for all participants.

Last, the groups were not balanced by race and sex, which could have led to signal quality differences across groups given that hair thickness, hair type, hair length, and skin pigmentation vary across these dimensions and that these variables are known to impact signal quality in fNIRS. When possible, future work should balance comparison groups by age and sex.

## 9 Conclusions and Future Directions

In sum, this work provides promising preliminary evidence of differentiation of cortical activity in bilateral fronto-temporoparietal regions for language formulation in discourse production tasks versus more purely speech production tasks using fNIRS. In addition, there is evidence of differences between three groups—young neurotypical individuals, individuals with aphasia, and age-matched neurotypical individuals. Use of novel discourse production paradigms allowed for evaluation of cortical activity with greater ecological validity than many traditional fMRI studies of language.

These two experiments provide compelling preliminary evidence for continued use of fNIRS as a tool for investigating task-based cortical activation in individuals with chronic post-stroke aphasia. From a clinical research standpoint, fNIRS could be used to assess a variety of discourse-level language tasks, both in production and comprehension in order to elucidate the cortical mechanisms underlying discourse production in neurotypical individuals and those with post-stroke aphasia. In addition, fNIRS may be suitable in the future to evaluate cortical activity during therapy tasks (e.g., which regions are engaged for tasks with varying cognitive-linguistic and pragmatic demands), cortical activity in real-world settings (e.g., in conversation at home or in the community), and changes in cortical activity over the course of stroke and aphasia recovery.

## Supplementary Material

Supplementary Material

## Data Availability

Raw data and statistical analysis code for this study are available on Open Science Framework (https://osf.io/nfevb/overview?view_only=b66f695bcfb94f37a8a7aaf437b51630).

## Footnotes

1 Normative sample t-score average = 50, SD = 10. Normed t-scores are specific to a participant’s age and education level.

2 ITK-SNAP completes automated lesion segmentation and allows for manual editing as necessary.

3 This included two channels of data for one age-matched participant across all runs due to known equipment malfunction during data acquisition (5% of long channels for this participant); one stimulus marker for one individual with aphasia (4% of stimulus markers for this participant); and one time block in each of two participants with aphasia which did not affect stimulus markers.

4 Normative sample t-score average = 50, SD = 10. Normed t-scores are specific to a participant’s age and education level.

## Appendix A2. Experiment 1 Example of Response for One Stimulus Item in a Young Neurotypical Participant

*Question:* Tell me about your favorite sport.

*Answer:* I like to play basketball. And I also like ping-pong. I think that it’s fun to do with friends or by yourself.
