# Supplementary material for: Cortical activity for conversational responses in young neurotypical individuals, older neurotypical individuals, and individuals with aphasia: A functional near-infrared spectroscopy study
Source: Imaging Neurosci (Camb). 2026 Feb 10;4:IMAG.a.1125. doi: 10.1162/IMAG.a.1125 (PMC12892348; doi:10.1162/IMAG.a.1125)
Supplement: Supplementary Material [file IMAG.a.1125_supp.pdf]

Supplemental Material

Experiment 1 Stimuli

Question Type	Block	Number	Question
Personal	1	1	Tell me about a T.V. show you like to watch.
		2	What do you like to do to relax?
		3	Tell me about your favorite music.
	2	1	What is your favorite season and why?
		2	Tell me about a place you've always wanted to visit.
		3	What kind of movies do you like to watch and why?
	3	1	What is your favorite holiday and why?
		2	Tell me about your favorite dessert.
		3	In your dream house, what is one special room you would include?
	4	1	Tell me about a food you don't like to eat.
		2	What do you like to do in the summertime?
		3	What country would you like to visit and why?
	5	1	What is something you have always wanted to learn?
		2	What is an ideal Sunday like for you?
		3	If you won the lottery, what would you plan to do with the money?
	6	1	Tell me about your favorite sport.
		2	Tell me about a hobby you have.
		3	If you could do any job, what would it be and why?
General	1	1	What do people do at the beach?
		2	What happens if your alarm doesn't go off in the morning?
		3	What are the Olympics?
	2	1	Who was George Washington?
		2	Tell me some things you can do using the internet.
		3	How do you plan a surprise party?
	3	1	What makes a good neighbor?
		2	What do people do for fun during the winter?
		3	How do you avoid dental cavities?
	4	1	Why do people have pets?
		2	How do Americans celebrate Thanksgiving?
		3	What do plants need to survive?
	5	1	Why do we exercise?
		2	What should you do if you hurt someone's feelings?
		3	What happens during a blizzard?
	6	1	What makes a good gift?
		2	What do people bring on a camping trip?
		3	How do we keep ourselves healthy?

fNIRS Probe Channel Coordinates and Location

Source	Detector	Channel Coordinates (MNI)	Label Name Per AtlasViewer	ROI
3	1	-36 17 24	Frontal_Inf_Oper_L	Frontal_Left
5	7	-58 21 16	Frontal_Inf_Oper_L	
1	5	-33 28 18	Frontal_Inf_Tri_L	
1	7	-45 26 21	Frontal_Inf_Tri_L	
1	1	-52 24 37	Frontal_Mid_L	
1	3	-42 13 28	Frontal_Inf_Oper_L	
3	5	-46 42 21	Frontal_Mid_L	
6	8	56 19 18	Frontal_Inf_Oper_R	Frontal_Right
2	4	48 16 31	Frontal_Inf_Oper_R	
2	8	46 24 19	Frontal_Inf_Tri_R	
2	6	54 36 21	Frontal_Mid_R	
2	2	51 23 36	Frontal_Mid_R	
4	2	49 34 42	Frontal_Mid_R	
4	6	48 40 19	Frontal_Mid_R	
9	9	-41 -26 26	SupraMarginal_L	Parietal_Left
9	11	-61 -29 43	SupraMarginal_L	
15	11	-40 -47 32	Angular_L	
15	9	-54 -54 29	Angular_L	
10	10	48 -34 24	SupraMarginal_R	Parietal_Right
10	12	50 -32 39	SupraMarginal_R	
16	12	63 -56 42	Parietal_Inf_R	
16	10	50 -48 25	Angular_R	
5	3	-56 12 31	Precentral_L	Precentral_Left
8	1	-43 7 55	Precentral_L	
8	3	-51 2 54	Precentral_L	
6	4	56 12 33	Precentral_R	Precentral_Right
7	4	40 -2 45	Precentral_R	
7	2	37 7 48	Precentral_R	
13	13	-45 -46 0	Temporal_Mid_L	Temporal_Left
13	9	-43 -47 16	Temporal_Mid_L	
11	9	-50 -30 11	Temporal_Mid_L	
11	13	-71 -32 2	Temporal_Mid_L	
12	14	49 -31 0	Temporal_Mid_R	Temporal_Right
12	10	46 -35 18	Temporal_Sup_R	
14	10	45 -42 18	Temporal_Sup_R	
14	14	51 -40 2	Temporal_Mid_R	

Experiment 1 Processing Stream Parameters

hmrR_PruneChannels	dRange	1e-03 1e+07
	SNRthresh	5
	SDrange	0.0 45.0
hmrR_Intensity2OD		
hmrR_MotionCorrectSplineSG	p	0.99
	FrameSize_sec	10
	turnon	1
hmrR_BandpassFilt: Bandpass_Filter_OpticalDensity	hpf	0.000
	lpf	0.500
hmrR_OD2Conc	ppf	1.0 1.0
hmrR_GLM	trange	-2.0 55.0
	glmSolveMethod	1
	idxBasis	1
	paramsBasis	1.0 1.0 0.0 0.0
	rhoSD_ssThresh	15.0
	flagNuisanceRMethod	1
	driftOrder	3
	c_vector	0

This is a screenshot of the fNIRS processing stream steps with specific parameters used in Homer3 with the following steps: 1) pruning channels with low signal to noise ratio; 2) conversion of raw data to optical density; 3) motion correction using a spline interpolation method; 4) low pass filtering; 5) conversation of optical density to concentration units; and 6) estimation of a generalized linear model for changes in HbO and HbR.

Experiment 1 Supplemental Figure

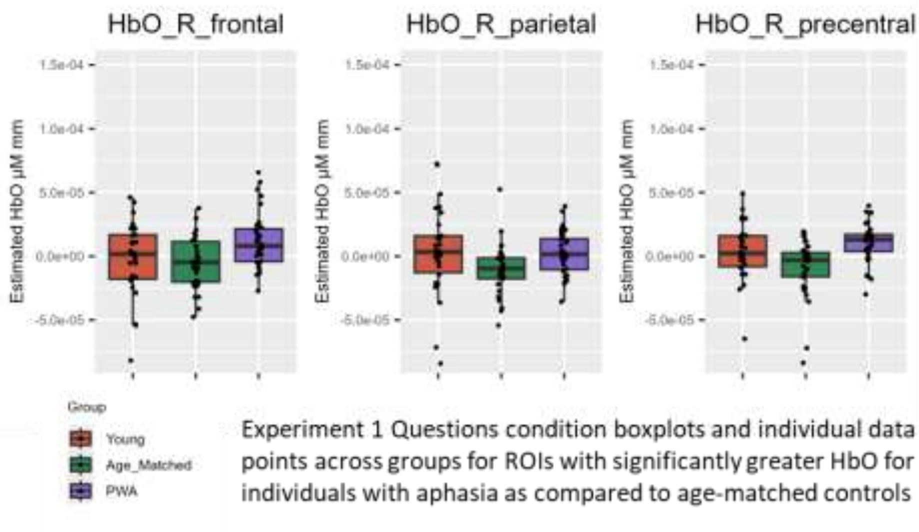

Experiment 1 Questions condition boxplots and individual data points across groups for ROIs with significantly greater HbO for individuals with aphasia as compared to age-matched controls

Experiment 1 Statistical Output

Experiment 1 Question 1 – Young Neurotypical

Dependent Variable	Independent Variables	Estimate	Std. Error	t value	p-value (uncorrected)	p-value (FDR corrected)
Left frontal	ConditionQuestions	0.00002026	0.00000582	3.48	0.00082	0.00655
	Epoch2	0.00001151	0.00000566	2.03	0.04583	0.07857
	Epoch3	0.00000665	0.00000566	1.17	0.24402	0.27888
Left precentral	ConditionQuestions	0.00000102	0.00000601	0.17	0.86534	0.86534
	Epoch2	0.00001150	0.00000583	1.97	0.05254	0.08406
	Epoch3	0.00000729	0.00000583	1.25	0.21551	0.25862
Left temporal	ConditionQuestions	0.00003573	0.00000596	6.00	0.00000	0.00000
	Epoch2	0.00001641	0.00000566	2.90	0.00496	0.01982
	Epoch3	0.00001742	0.00000566	3.08	0.00295	0.01418
Left parietal	ConditionQuestions	0.00001625	0.00000614	2.65	0.00988	0.02965
	Epoch2	0.00001234	0.00000580	2.13	0.03678	0.06790
	Epoch3	0.00000951	0.00000580	1.64	0.10539	0.14879
Right frontal	ConditionQuestions	0.00001517	0.00000625	2.43	0.01742	0.04180
	Epoch2	0.00000955	0.00000610	1.57	0.12170	0.16227
	Epoch3	0.00000663	0.00000610	1.09	0.28047	0.30596
Right precentral	ConditionQuestions	-0.00001419	0.00000813	-1.74	0.08465	0.12697
	Epoch2	0.00001107	0.00000866	1.28	0.20463	0.25848
	Epoch3	0.00000551	0.00000866	0.64	0.52595	0.54882
Right temporal	ConditionQuestions	0.00002358	0.00000543	4.34	0.00004	0.00051
	Epoch2	0.00001446	0.00000523	2.77	0.00721	0.02473
	Epoch3	0.00001177	0.00000523	2.25	0.02742	0.05484
Right parietal	ConditionQuestions	0.00001428	0.00000602	2.37	0.02019	0.04405
	Epoch2	0.00001818	0.00000575	3.16	0.00229	0.01372
	Epoch3	0.00001497	0.00000575	2.61	0.01115	0.02974

Experiment 1 Question 1 – Age-Matched Neurotypical

Dependent Variable	Independent Variables	Estimate	Std. Error	t value	p-value (uncorrected)	p-value (FDR corrected)
Left frontal	ConditionQuestions	0.00001198	0.00000662	1.81	0.07442	0.21927
	Epoch2	0.00001176	0.00000692	1.70	0.09423	0.21927
	Epoch3	0.00000990	0.00000692	1.43	0.15756	0.28512
Left precentral	ConditionQuestions	0.00000783	0.00000461	1.70	0.09360	0.21927
	Epoch2	0.00000660	0.00000471	1.40	0.16632	0.28512
	Epoch3	0.00000542	0.00000471	1.15	0.25453	0.38062
Left temporal	ConditionQuestions	0.00001423	0.00000721	1.97	0.05227	0.20910
	Epoch2	0.00000823	0.00000740	1.11	0.26961	0.38062
	Epoch3	0.00001044	0.00000740	1.41	0.16270	0.28512
Left parietal	ConditionQuestions	0.00001742	0.00000614	2.84	0.00588	0.07061
	Epoch2	0.00001356	0.00000641	2.12	0.03805	0.18262
	Epoch3	0.00001432	0.00000641	2.23	0.02886	0.18262
Right frontal	ConditionQuestions	0.00000384	0.00000564	0.68	0.49760	0.59712
	Epoch2	0.00000775	0.00000589	1.32	0.19257	0.30811
	Epoch3	0.00001252	0.00000589	2.13	0.03724	0.18262
Right precentral	ConditionQuestions	0.00000280	0.00000652	0.43	0.66898	0.69807
	Epoch2	0.00000322	0.00000718	0.45	0.65507	0.69807
	Epoch3	0.00000514	0.00000718	0.72	0.47613	0.59712
Right temporal	ConditionQuestions	0.00000008	0.00000631	0.01	0.98984	0.98984
	Epoch2	0.00000640	0.00000675	0.95	0.34693	0.46257
	Epoch3	0.00001245	0.00000675	1.84	0.06986	0.21927
Right parietal	ConditionQuestions	0.00000225	0.00000489	0.46	0.64714	0.69807
	Epoch2	0.00000843	0.00000506	1.67	0.10050	0.21927
	Epoch3	0.00001453	0.00000506	2.87	0.00551	0.07061

Experiment 1 Question 1 – Individuals with Aphasia

Dependent Variable	Independent Variables	Estimate	Std. Error	t value	p-value (uncorrected)	p-value (FDR corrected)
Left frontal	ConditionQuestions	-0.00001610	0.00000844	-1.91	0.06166	0.44138
	Epoch2	0.00000800	0.00000882	0.91	0.36871	0.64238
	Epoch3	0.00000510	0.00000882	0.58	0.56588	0.75450
Left precentral	ConditionQuestions	-0.00001033	0.00000604	-1.71	0.09195	0.44138
	Epoch2	0.00001225	0.00000689	1.78	0.08052	0.44138
	Epoch3	0.00000932	0.00000689	1.35	0.18123	0.54369
Left temporal	ConditionQuestions	0.00000669	0.00000784	0.85	0.39821	0.64238
	Epoch2	0.00001806	0.00000907	1.99	0.05374	0.44138
	Epoch3	0.00000769	0.00000907	0.85	0.40149	0.64238
Left parietal	ConditionQuestions	-0.00000292	0.00000555	-0.53	0.60056	0.75860
	Epoch2	0.00001268	0.00000588	2.16	0.03632	0.44138
	Epoch3	0.00000582	0.00000588	0.99	0.32732	0.64238
Right frontal	ConditionQuestions	-0.00000435	0.00000442	-0.98	0.32828	0.64238
	Epoch2	0.00000120	0.00000457	0.26	0.79359	0.86719
	Epoch3	-0.00000010	0.00000457	-0.02	0.98177	0.98177
Right precentral	ConditionQuestions	-0.00000293	0.00000372	-0.79	0.43341	0.65011
	Epoch2	0.00000098	0.00000425	0.23	0.81857	0.86719
	Epoch3	-0.00000281	0.00000425	-0.66	0.50992	0.71989
Right temporal	ConditionQuestions	-0.00000379	0.00000381	-0.99	0.32036	0.64238
	Epoch2	0.00000661	0.00000420	1.57	0.11593	0.46373
	Epoch3	0.00000097	0.00000420	0.23	0.81805	0.86719
Right parietal	ConditionQuestions	-0.00000410	0.00000396	-1.04	0.30327	0.64238
	Epoch2	0.00000661	0.00000452	1.46	0.14682	0.50339
	Epoch3	0.00000097	0.00000452	0.21	0.83105	0.86719

Experiment 1 Question 2

Dependent Variable	Independent Variables	Estimate	Std. Error	t value	p-value (uncorrected)	p-value (FDR corrected)
Left frontal	ConditionQuestions	0.00000723	0.00000701	1.03	0.30722	0.46083
	GroupYoung	0.00000427	0.00000953	0.45	0.65619	0.71967
	GroupPWA	0.00000565	0.00001026	0.55	0.58486	0.70184
Left precentral	ConditionQuestions	-0.00000080	0.00000527	-0.15	0.87960	0.87960
	GroupYoung	-0.00000131	0.00000809	-0.16	0.87181	0.87960
	GroupPWA	0.00001237	0.00000873	1.42	0.16409	0.32818
Left temporal	ConditionQuestions	0.00002126	0.00000671	3.17	0.00282	0.03286
	GroupYoung	0.00003677	0.00001203	3.06	0.00411	0.03286
	GroupPWA	0.00002598	0.00001365	1.90	0.06497	0.17326
Left parietal	ConditionQuestions	0.00001223	0.00000558	2.19	0.03397	0.11648
	GroupYoung	0.00001789	0.00001241	1.44	0.15736	0.32818
	GroupPWA	0.00001839	0.00001383	1.33	0.19139	0.35333
Right frontal	ConditionQuestions	0.00000503	0.00000485	1.04	0.30410	0.46083
	GroupYoung	0.00000467	0.00000755	0.62	0.53904	0.68089
	GroupPWA	0.00001877	0.00000735	2.55	0.01419	0.06810
Right precentral	ConditionQuestions	-0.00000524	0.00000555	-0.95	0.34732	0.49033
	GroupYoung	0.00001349	0.00000656	2.05	0.04302	0.12906
	GroupPWA	0.00002252	0.00000637	3.54	0.00066	0.01573
Right temporal	ConditionQuestions	0.00000874	0.00000540	1.62	0.11137	0.26728
	GroupYoung	0.00000838	0.00000782	1.07	0.28988	0.46083
	GroupPWA	0.00000337	0.00000761	0.44	0.65970	0.71967
Right parietal	ConditionQuestions	0.00000311	0.00000466	0.67	0.50665	0.67554
	GroupYoung	0.00001965	0.00000770	2.55	0.01413	0.06810
	GroupPWA	0.00001769	0.00000750	2.36	0.02281	0.09123

Experiment 1 Results – HbR

Research Question 1: In three groups (young neurotypical individuals, individuals with post-stroke aphasia, and age-matched neurotypical individuals), can functional near-infrared spectroscopy (fNIRS) be used to index cortical activity differences between language formulation (i.e., conversational responses) and sentence repetition?

For the young neurotypical group before multiple comparison correction, there was a significant effect of condition for HbR in the left parietal and right precentral ROIs (questions > repeat, all $p < .05$). There were no significant effects after multiple comparison correction.

For the age-matched neurotypical group before multiple comparison correction, there was a significant effect of epoch for HbR in the left frontal ROI (Epoch 3 > Epoch 1, $p = .034$). There were no significant effects after multiple comparison correction.

For the individuals with aphasia before multiple comparison correction, there was a significant effect of condition for HbR in the left precentral ROI (questions > repeat, $p = .031$) and a significant effect of epoch in the left precentral ROI (Epoch 3 < Epoch 1, $p = .021$). There were no significant effects after multiple comparison correction.

Research Question 2: Are there differences in cortical activity for conversational responses and sentence repetition among young neurotypical individuals, individuals with post-stroke aphasia, and age-matched neurotypical individuals?

Before multiple comparison correction, there was a significant effect of group for HbR in the right parietal ROI (young > age-matched, $p = .034$). There were no significant effects after multiple comparison correction.

Experiment 1 Results – Interaction Models (HbO and HbR)

Research Question 1: In three groups (young neurotypical individuals, individuals with post-stroke aphasia, and age-matched neurotypical individuals), can functional near-infrared spectroscopy (fNIRS) be used to index cortical activity differences between language formulation (i.e., conversational responses) and sentence repetition?

For the young neurotypical group in the interaction models before multiple comparison correction, there was an interaction for HbO in the left temporal ROI for condition x Epoch 2 ($p = .024$) and condition x Epoch 3 ($p = .026$). Follow-up pairwise comparisons showed significantly higher HbO for questions vs. repeat at Epoch 2 ($p < .001$) and Epoch 3 ($p < .001$) but not at Epoch 1 ($p = .518$). Furthermore, there was significantly higher HbO for questions at Epoch 2 vs. Epoch 1 ($p = .002$) and at Epoch 3 vs. Epoch 1 ($p = .001$) but not for repeat at Epoch 2 vs. Epoch 1 ($p = .966$) or Epoch 3 vs. Epoch 1 ($p = .93$). In other words, HbO in the left temporal ROI increased across epochs for the questions condition but not the repeat condition. For the young neurotypical group in the interaction model after multiple comparison correction, there were no significant interactions (all $p > .05$). For the HbR interaction models, there were no significant condition x epoch interactions before or after multiple comparison correction.

For the age-matched neurotypical group and the individuals with aphasia in the interaction models, there were no significant interaction effects for HbO or HbR across ROIs before or after multiple comparison correction (all $p > .05$).

Research Question 2: Are there differences in cortical activity for conversational responses and sentence repetition among young neurotypical individuals, individuals with post-stroke aphasia, and age-matched neurotypical individuals?

In the interaction models, there were no significant group x condition interactions for HbO or HbR in any ROIs before or after multiple comparison correction (all $p > .05$).

Experiment 2 Stimuli

Question Type	Run	Question
Personal	1	Tell me about a T.V. show you like to watch.
		Tell me about your favorite music.
		What is your favorite season and why?
		Tell me about a place you've always wanted to visit.
		Tell me about your favorite dessert.
		What is your favorite holiday and why?
	2	Tell me about a food you don't like to eat.
		What do you like to do in the summertime?
		What country would you like to visit and why?
		What is something you have always wanted to learn?
		Tell me about a hobby you have.
		What is an ideal Sunday like for you?
General	1	What do people do at the beach?
		What are the Olympics?
		What makes a good neighbor?
		What do people do for fun during the winter?
		How do you avoid dental cavities?
		How do you plan a surprise party?
	2	Why do people have pets?
		Why do we exercise?
		What happens during a blizzard?
		What makes a good gift?
		How do we keep ourselves healthy?
		What should you do if you hurt someone's feelings?

Experiment 2 Processing Stream Parameters

hmrR_PruneChannels	dRange	1e-03 1e+07
	SNRthresh	5
	SDrange	0.0 45.0
hmrR_Intensity2OD		
hmrR_MotionCorrectSplineSG	p	0.99
	FrameSize_sec	10
	turnon	1
hmrR_BandpassFilt: Bandpass_Filter_OpticalDensity	hpf	0.000
	lpf	0.500
hmrR_OD2Conc	ppf	1.0 1.0
hmrR_GLM	trange	-2.0 25.0
	glmSolveMethod	1
	idxBasis	1
	paramsBasis	1.0 1.0 0.0 0.0
	rhoSD_ssThresh	15.0
	flagNuisanceRMethod	1
	driftOrder	3
	c_vector	0

This is a screenshot of the fNIRS processing stream steps with specific parameters used in Homer3 with the following steps: 1) pruning channels with low signal to noise ratio; 2) conversion of raw data to optical density; 3) motion correction using a spline interpolation method; 4) low pass filtering; 5) conversation of optical density to concentration units; and 6) estimation of a generalized linear model for changes in HbO and HbR.

Experiment 2 Statistical Output

Experiment 2 Question 1 – Young Neurotypical

Dependent Variable	Independent Variables	Estimate	Std. Error	t value	p-value (uncorrected)	p-value (FDR corrected)
Left frontal	ConditionAnswer	0.00000351	0.00000534	0.66	0.51292	0.71363
	Epoch2	0.00000482	0.00000481	1.00	0.31928	0.63857
	Epoch3	-0.00000265	0.00000481	-0.55	0.58384	0.77845
	Epoch4	0.00000165	0.00000481	0.34	0.73231	0.90130
Left temporal	ConditionAnswer	-0.00000009	0.00000831	-0.01	0.99094	0.99449
	Epoch2	0.00000919	0.00000748	1.23	0.22198	0.52042
	Epoch3	0.00001157	0.00000748	1.55	0.12501	0.52042
	Epoch4	0.00002035	0.00000748	2.72	0.00768	0.12287
Left parietal	ConditionAnswer	0.00000520	0.00000762	0.68	0.49645	0.71363
	Epoch2	0.00000105	0.00000671	0.16	0.87551	0.93387
	Epoch3	-0.00000909	0.00000671	-1.36	0.17841	0.52042
	Epoch4	0.00001425	0.00000671	2.12	0.03613	0.28902
Left precentral	ConditionAnswer	0.00000830	0.00000577	1.44	0.15290	0.52042
	Epoch2	-0.00000150	0.00000499	-0.30	0.76475	0.90637
	Epoch3	-0.00001539	0.00000499	-3.08	0.00266	0.08510
	Epoch4	-0.00000381	0.00000499	-0.76	0.44789	0.68459
Right frontal	ConditionAnswer	0.00000443	0.00000565	0.78	0.43467	0.68459
	Epoch2	0.00000473	0.00000620	0.76	0.44727	0.68459
	Epoch3	-0.00000516	0.00000620	-0.83	0.40705	0.68459
	Epoch4	-0.00000266	0.00000620	-0.43	0.66899	0.85631
Right temporal	ConditionAnswer	0.00000959	0.00000744	1.29	0.20024	0.52042
	Epoch2	-0.00000166	0.00000636	-0.26	0.79506	0.90864
	Epoch3	-0.00001163	0.00000636	-1.83	0.07047	0.39107
	Epoch4	0.00000806	0.00000636	1.27	0.20790	0.52042
Right parietal	ConditionAnswer	0.00000634	0.00000835	0.76	0.44926	0.68459
	Epoch2	-0.00000005	0.00000726	-0.01	0.99449	0.99449
	Epoch3	-0.00001313	0.00000726	-1.81	0.07333	0.39107
	Epoch4	0.00000898	0.00000726	1.24	0.21896	0.52042
Right precentral	ConditionAnswer	0.00000644	0.00000533	1.21	0.22768	0.52042
	Epoch2	0.00000483	0.00000458	1.05	0.29340	0.62591
	Epoch3	-0.00001078	0.00000458	-2.35	0.01944	0.20734
	Epoch4	0.00000076	0.00000458	0.17	0.86853	0.93387

Experiment 1 Question 1 – Age-Matched Neurotypical

Dependent Variable	Independent Variables	Estimate	Std. Error	t value	p-value (uncorrected)	p-value (FDR corrected)
Left frontal	ConditionAnswer	0.00000811	0.00000730	1.11	0.26964	0.47937
	Epoch2	0.00000099	0.00000582	0.17	0.86486	0.89276
	Epoch3	-0.00001340	0.00000582	-2.30	0.02335	0.06227
	Epoch4	-0.00002046	0.00000582	-3.52	0.00066	0.00529
Left temporal	ConditionAnswer	-0.00000213	0.00000628	-0.34	0.73608	0.89276
	Epoch2	0.00000900	0.00000554	1.63	0.10721	0.22872
	Epoch3	-0.00000442	0.00000554	-0.80	0.42622	0.64948
	Epoch4	-0.00001180	0.00000554	-2.13	0.03538	0.08709
Left parietal	ConditionAnswer	0.00000217	0.00000698	0.31	0.75658	0.89276
	Epoch2	0.00000109	0.00000515	0.21	0.83226	0.89276
	Epoch3	-0.00002011	0.00000515	-3.90	0.00017	0.00274
	Epoch4	-0.00001832	0.00000515	-3.56	0.00057	0.00529
Left precentral	ConditionAnswer	0.00000586	0.00000601	0.97	0.33125	0.55789
	Epoch2	0.00000161	0.00000453	0.36	0.72228	0.89276
	Epoch3	-0.00001295	0.00000453	-2.86	0.00465	0.02181
	Epoch4	-0.00001330	0.00000453	-2.93	0.00366	0.02181
Right frontal	ConditionAnswer	-0.00000127	0.00000621	-0.21	0.83779	0.89276
	Epoch2	0.00000390	0.00000474	0.82	0.41301	0.64948
	Epoch3	-0.00001322	0.00000474	-2.79	0.00638	0.02553
	Epoch4	-0.00002302	0.00000474	-4.85	0.00000	0.00015
Right temporal	ConditionAnswer	0.00000022	0.00000684	0.03	0.97400	0.97400
	Epoch2	0.00000728	0.00000537	1.36	0.17797	0.33501
	Epoch3	-0.00000335	0.00000537	-0.62	0.53350	0.74187
	Epoch4	-0.00000837	0.00000537	-1.56	0.12223	0.24447
Right parietal	ConditionAnswer	0.00000101	0.00000559	0.18	0.85750	0.89276
	Epoch2	0.00000984	0.00000511	1.92	0.05709	0.13050
	Epoch3	-0.00001236	0.00000511	-2.42	0.01747	0.05082
	Epoch4	-0.00001476	0.00000511	-2.89	0.00477	0.02181
Right precentral	ConditionAnswer	0.00000369	0.00000626	0.59	0.55640	0.74187
	Epoch2	0.00000276	0.00000447	0.62	0.53765	0.74187
	Epoch3	-0.00001205	0.00000447	-2.69	0.00766	0.02575
	Epoch4	-0.00001197	0.00000447	-2.68	0.00805	0.02575

Experiment 1 Question 1 – People with Aphasia

Dependent Variable	Independent Variables	Estimate	Std. Error	t value	p-value (uncorrected)	p-value (FDR corrected)
Left frontal	ConditionAnswer	0.00000720	0.00000723	1.00	0.32286	0.60753
	Epoch2	0.00000454	0.00000788	0.58	0.56673	0.75564
	Epoch3	0.00000130	0.00000788	0.17	0.86919	0.89747
	Epoch4	0.00000102	0.00000788	0.13	0.89747	0.89747
Left temporal	ConditionAnswer	0.00002176	0.00000700	3.11	0.00268	0.06894
	Epoch2	0.00001702	0.00000760	2.24	0.02843	0.13424
	Epoch3	0.00001582	0.00000760	2.08	0.04114	0.16457
	Epoch4	0.00001911	0.00000760	2.52	0.01432	0.11458
Left parietal	ConditionAnswer	0.00001446	0.00000649	2.23	0.02937	0.13424
	Epoch2	0.00000753	0.00000735	1.02	0.31001	0.60753
	Epoch3	-0.00000237	0.00000735	-0.32	0.74881	0.89685
	Epoch4	0.00000196	0.00000735	0.27	0.79081	0.89685
Left precentral	ConditionAnswer	0.00000476	0.00000499	0.95	0.34174	0.60753
	Epoch2	0.00000544	0.00000536	1.02	0.31203	0.60753
	Epoch3	-0.00000128	0.00000536	-0.24	0.81106	0.89685
	Epoch4	0.00000321	0.00000536	0.60	0.55079	0.75564
Right frontal	ConditionAnswer	-0.00000426	0.00000348	-1.22	0.22351	0.51088
	Epoch2	0.00001224	0.00000472	2.59	0.01082	0.11458
	Epoch3	0.00000632	0.00000472	1.34	0.18320	0.45096
	Epoch4	-0.00000112	0.00000472	-0.24	0.81277	0.89685
Right temporal	ConditionAnswer	0.00000950	0.00000428	2.22	0.02860	0.13424
	Epoch2	0.00000949	0.00000582	1.63	0.10589	0.33884
	Epoch3	-0.00000280	0.00000582	-0.48	0.63211	0.80910
	Epoch4	-0.00000347	0.00000582	-0.60	0.55253	0.75564
Right parietal	ConditionAnswer	0.00000473	0.00000339	1.40	0.16536	0.44095
	Epoch2	0.00001343	0.00000461	2.92	0.00431	0.06894
	Epoch3	-0.00000408	0.00000461	-0.89	0.37767	0.62666
	Epoch4	-0.00000670	0.00000461	-1.46	0.14852	0.43205
Right precentral	ConditionAnswer	-0.00000051	0.00000348	-0.15	0.88410	0.89747
	Epoch2	0.00000907	0.00000477	1.90	0.05966	0.21214
	Epoch3	0.00000393	0.00000477	0.82	0.41124	0.62666
	Epoch4	0.00000405	0.00000477	0.85	0.39740	0.62666

Experiment 2 Question 2

Dependent Variable	Independent Variables	Estimate	Std. Error	t value	p-value (uncorrected)	p-value (FDR corrected)
Left frontal	ConditionQuestions	0.00000408	0.00000765	0.53	0.59584	0.71501
	GroupYoung	0.00000792	0.00000891	0.89	0.37960	0.71501
	GroupPWA	0.00000631	0.00000838	0.75	0.45603	0.71501
Left precentral	ConditionQuestions	0.00000957	0.00000738	1.30	0.19836	0.71501
	GroupYoung	0.00000812	0.00000750	1.08	0.28255	0.71501
	GroupPWA	-0.00000673	0.00000712	-0.95	0.34719	0.71501
Left temporal	ConditionQuestions	0.00000462	0.00000826	0.56	0.57773	0.71501
	GroupYoung	0.00001343	0.00000919	1.46	0.15186	0.71501
	GroupPWA	0.00000665	0.00000867	0.77	0.44749	0.71501
Left parietal	ConditionQuestions	0.00000479	0.00000739	0.65	0.51977	0.71501
	GroupYoung	0.00000947	0.00001085	0.87	0.38829	0.71501
	GroupPWA	0.00000531	0.00000980	0.54	0.59068	0.71501
Right frontal	ConditionQuestions	-0.00000100	0.00000662	-0.15	0.87979	0.95977
	GroupYoung	0.00001106	0.00000687	1.61	0.11129	0.71501
	GroupPWA	0.00000017	0.00000733	0.02	0.98176	0.98383
Right precentral	ConditionQuestions	0.00000282	0.00000594	0.47	0.63634	0.72725
	GroupYoung	0.00001046	0.00000617	1.70	0.09338	0.71501
	GroupPWA	0.00000366	0.00000658	0.56	0.57950	0.71501
Right temporal	ConditionQuestions	0.00000563	0.00000501	1.13	0.26561	0.71501
	GroupYoung	0.00000657	0.00001002	0.66	0.51586	0.71501
	GroupPWA	-0.00001122	0.00001037	-1.08	0.28488	0.71501
Right parietal	ConditionQuestions	0.00000013	0.00000664	0.02	0.98383	0.98383
	GroupYoung	0.00000576	0.00000689	0.84	0.40562	0.71501
	GroupPWA	-0.00000515	0.00000735	-0.70	0.48566	0.71501

Experiment 2 Results – HbR

Research Question 1: **In three groups (young neurotypical individuals, individuals with post-stroke aphasia, and age-matched neurotypical individuals), can functional near-infrared spectroscopy (fNIRS) be used to index cortical activity differences between language formulation (i.e., conversational responses) and sentence repetition?**

For the young neurotypical group before multiple comparison correction, there was a significant effect of condition for HbR in the left temporal and right temporal ROIs (answer > repeat; all $p < .05$). There was a significant effect of epoch in the left frontal (Epoch 3 < Epoch 1), right frontal (Epoch 3 < HbR Epoch 1), and right parietal (Epoch 4 > Epoch 1) ROIs (all $p < .05$). After multiple comparison correction, there remained a significant effect of condition in the left temporal ROI (answer > repeat; $p < .001$).

For the age-matched neurotypical group before multiple comparison correction, there was a significant effect of condition for HbR in the left frontal ROI (answer > repeat; $p = .035$). There was a significant effect of epoch in the left frontal (Epoch 3 < Epoch 1; Epoch 4 < Epoch 1), left temporal (Epoch 3 < Epoch 1), left parietal (Epoch 2 < Epoch 1; Epoch 3 < Epoch 1; Epoch 4 < Epoch 1), right parietal (Epoch 3 < Epoch 1; Epoch 4 < Epoch 1), and right precentral (Epoch 3 < Epoch 1; Epoch 4 < Epoch 1) ROIs (all $p < .05$). After multiple comparison correction, there remained a significant effect of condition in the left frontal (Epoch 3 < Epoch 1), left parietal (Epoch 3 < Epoch 1), right parietal (Epoch 3 < Epoch 1), and right precentral (Epoch 3 < Epoch 1; Epoch 4 < Epoch 1) ROIs (all $p < .05$).

For the individuals with aphasia before multiple comparison correction, there was a significant effect of condition for HbR in the left temporal, left parietal, right temporal, right parietal, and right precentral ROIs (answer > repeat; all $p < .05$). There was a significant effect of epoch in the right precentral ROI (Epoch 4 < Epoch 1, $p = .006$). After multiple comparison correction, there remained a significant effect or condition in the right temporal ROI (answer > repeat, $p = .021$).

Research Question 2: **Are there differences in cortical activity for conversational responses and sentence repetition among young neurotypical individuals, individuals with post-stroke aphasia, and age-matched neurotypical individuals?**

Before multiple comparison correction, there was a significant effect of condition for HbR in the left temporal ROI (answer > repeat; $p < .001$) as well as a significant effect of group in the left temporal ROI (PWA > age-matched; $p = .009$). There was also a significant effect of condition in the right temporal ROI (answer > repeat; $p = .038$) and a significant effect of group in the right temporal ROI (PWA > age-matched; $p = .017$). After multiple comparison correction, there remained a significant effect of condition in the left temporal ROI (answer > repeat; $p = .015$).

Experiment 2 Results – Interaction Models (HbO and HbR)

Research Question 1: **In three groups (young neurotypical individuals, individuals with post-stroke aphasia, and age-matched neurotypical individuals), can functional near-infrared spectroscopy (fNIRS) be used to index cortical activity differences between language formulation (i.e., conversational responses) and sentence repetition?**

For the young neurotypical group in the HbO interaction models before multiple comparison correction, there was a significant condition x Epoch 4 interaction for HbO in the left frontal ROI ($p = .036$). However, follow-up pairwise comparisons showed no significant differences between answer and repeat at Epoch 1 or 4 or between repeat at Epoch 1 vs. repeat at Epoch 4 or answer at Epoch 1 vs. answer at Epoch 4 after multiple comparison correction. For the young neurotypical group in the interaction models after multiple comparison correction, there were no significant condition x epoch interactions (all adjusted $p > .05$). For the young neurotypical group in the HbR interaction models, there were no significant condition x epoch interactions before or after multiple comparison correction.

For the age-matched neurotypical group in the interaction models before multiple comparison correction, there was a significant condition x Epoch 4 interaction for HbO in the left temporal, right temporal, and right parietal ROIs (all $p < .05$). In follow-up pairwise comparisons in the left temporal ROI, there was significantly decreased HbO in the repeat condition from Epoch 1 to Epoch 4 ($p = .003$) while there was not significantly decreased HbO on the answer condition from Epoch 1 to Epoch 4 ($p = .975$). In follow-up pairwise comparisons in the right temporal ROI, the same pattern was seen, with significantly decreased HbO in the repeat condition from Epoch 1 to Epoch 4 ($p = .027$), while there was not significantly decreased HbO in the answer condition from Epoch 1 to Epoch 4 ($p = .939$). In follow-up pairwise comparisons in the left parietal ROI, the same pattern was seen, with significantly decreased HbO in the repeat condition from Epoch 1 to Epoch 4 ($p = .004$) while there was not significantly decreased HbO in the answer condition from Epoch 1 to Epoch 4 ($p = .442$). For the age-matched neurotypical group in the interaction models after multiple comparison correction, there were no significant epoch x condition interactions for HbO across ROIs (all adjusted $p > .05$).

For the age-matched neurotypical group in the interaction models before multiple comparison correction, there was a significant interaction for condition x Epoch 4 for HbR in the right temporal ROI ($p = .044$) and significant condition x Epoch 3 ($p = .041$) and condition x Epoch 4 ($p = .034$) interactions in the right parietal ROI. Follow-up pairwise comparison showed no significant differences for HbR in the right temporal ROI after multiple comparison correction (all $p > .05$). Follow-up pairwise comparisons for the right parietal ROI showed lower HbR in the repeat condition at Epoch 4 vs. Epoch 1 ($p = .006$) but no difference in the answer condition at Epoch 4 vs. Epoch 1 ($p > .999$). For the age-matched neurotypical group in the interaction models after multiple comparison correction, there were no significant condition x epoch interactions for HbR across ROIs (all adjusted $p > .05$).

For the people with aphasia in the interaction models before multiple comparison correction, there was a significant interaction for condition x Epoch 3 interaction for HbO in the

left temporal ROI (p = .023). Follow-up pairwise comparison showed increase in HbO between Epoch 1 and Epoch 3 in the answer condition (p = .013), but no difference in the repeat condition between Epoch 1 and Epoch 3 (p = .999). For the people with aphasia in the interaction model after multiple comparison correction, there were no significant condition x epoch interactions for HbO across ROIs (all adjusted p > .05).

For the people with aphasia in the interaction models before multiple comparison correction, there was a significant interaction for condition x Epoch 3 for HbR in the left temporal ROI (p = .006). Follow-up pairwise comparisons showed greater HbR (less negative) in the answer condition for Epoch 3 vs. Epoch 1 (p = .01) but no difference in the repeat condition for Epoch 3 vs. Epoch 1 (p = .832). For the people with aphasia in the interaction models after multiple comparison correction, there were no significant condition x epoch interactions for HbR across ROIs (all adjusted p > .05).

***Research Question 2:* Are there differences in cortical activity for conversational responses and sentence repetition among young neurotypical individuals, individuals with post-stroke aphasia, and age-matched neurotypical individuals?**

In the interaction models, there were no significant group x condition effects for HbO or HbR across ROIs before or after multiple comparison correction (all p > .05).
